# Supplementary material for: Optimising acute stroke care organisation: a simulation study to assess the potential to increase intravenous thrombolysis rates and patient gains
Source: BMJ Open. 2020 Jan 20;10(1):e032780. doi: 10.1136/bmjopen-2019-032780 (PMC7045180; doi:10.1136/bmjopen-2019-032780)
Supplement: Supplementary data [file bmjopen-2019-032780supp001.pdf]

Supplementary file

Optimizing acute stroke care organization: a simulation study to assess the potential to increase intravenous thrombolysis rates and patient gains

Maarten M.H. Lahr Ph.D., Durk-Jouke van der Zee Ph.D., Gert-Jan Luijckx M.D., Ph.D., Erik Buskens M.D., Ph.D.

Introduction

The main body of text in the manuscript summarizes and discusses the most important results of the study. This supplementary file provides further details on the simulation modeling methodology that was used, the process map underlying the simulation model, model data, and input parameters characterizing scenarios studied.

Simulation modeling methodology

Monte Carlo simulation

The simulation model built conforms to the notion of Monte Carlo simulation.[1, 2] The model represents the acute stroke pathway as it evolves over time by a representation in which state variables change instantaneously at separate, i.e. discrete, points in time.[3] Variety in patient characteristics, activity durations, and medical decision making concerning diagnosis, and intravenous thrombolysis (IVT) treatment are incorporated into the model by probability distributions derived from real system (patient) data. Activity durations include possible patient queueing times, if any, as the stroke patients' urgency usually allows them to queue

jump, effectively rendering waiting times for intra hospital services to remain negligible . Moreover, given their low numbers stroke patients have very limited impact on queueing behavior along the pathway.[4]

Distribution fitting

Probability distributions associated with patient characteristics, and activity durations were determined (fitted) using ExpertFit[5] (Table S1). Main steps concerned:

- Importing real system (patient) data into ExpertFit.
- Fitting theoretical distributions by using the method of maximum likelihood.[3]
- Seeking further evidence in case of a "no fit", in an attempt to underpin the choice for a specific theoretical distribution. Evidence considered includes conceptual usage of the candidate distribution(s), commonalities between highest ranked distributions, and consultation of domain experts.[6] If such evidence is not found an empirical distribution was chosen.

Set-up of experiments

All experiments concern observations on 10,000 hypothetical patients. The number of patients is chosen such that the 95% confidence interval half width is below 1% of the mean treatment rate.

Software

Plant Simulation was used to model the stroke pathway.[7] Choice of probability distributions and their respective parameters is made using ExpertFit.[5]

Model - process map and data

Model set-up conforms to description of the stroke pathway (Main text, Figure 1). Further details are provided in the process map (Figure S1) and the overview of distributions of time delays and diagnostic characteristics (Table S1). All time delays are expressed in minutes.

Patients are classified according to their route, i.e., mode of transportation towards the hospital:

(1) Emergency Medical Services (EMS): Assumes patients being transported to the hospital by EMS. A patient can be in Route 1 with a probability of 76%. If the patient is in Route 1, then the following quantities need to be simulated for modelling pre-hospital activities: the time from symptom onset to call for help, the choice and time delay at the first responder (i.e. either the general practitioner or 911), the level of urgency set for EMS transport, the time between 911 activation and arrival of the ambulance at the location of the patient, the time spent by ambulance personnel at the location of the patient, and the time required to transport the patient to the hospital. Three levels of urgency for EMS transport are distinguished, i.e., A1, A2, and B. They indicate normative values for ambulance arrival within 15, 30, and > 30 minutes from the 911 call until arrival at the location of the patients. Urgency levels impact EMS response time, time spent on scene and transport time. Intrahospital activities assume the following quantities to be simulated: the time from hospital arrival to neurological examination, the time required for neuroimaging (Computed Tomography, CT scan), the time to laboratory examination of patient blood samples, the time to reach a decision on patient treatment, and the time it takes to mix thrombolytics.

A small group of patients (2% of overall population) in route 1 is initially transported to a community hospital offering no stroke services, thereby facing significant time losses. As

such patients never become eligible for treatment in the real system no quantities were simulated, except for their arrival at the Emergency Department (ED).

(2) Self-transport: Assumes patients not being transported to the hospital by EMS. Instead patients or family/bystanders take care of transportation. A patient can be in Route 2 with a probability of 21%. We simplified the model with respect to the inclusion of patients in Route 2. As none of these patients appeared eligible for treatment in the real system, no quantities were simulated, except for their arrival at the ED. Note how Table S1 clarifies that all patients in this route, except for two, who arrived way beyond the period of 4.5 hours after stroke onset, for whom thrombolysis treatment has been found to be effective.

(3) In-hospital patients: Patients suffering a stroke while being hospitalized. A patient can be in route 3 with a probability of 3%. If the patient is in Route 3 only intra-hospital time delays need to simulated, see route 1.

Next, traversing each route entails sampling from distributions specifying respective activity durations. Note how activity durations may be moderated by diagnostic outcomes. Finally, cumulative delay for a patient is used as an input for the treatment decision. Here a larger delay implies a smaller chance of being treated.

Timing of stroke onset on the day did not affect our model assumptions, because we did not expect any impact on capacity constraints. Out of office hours were not included because the hospital under study (University Medical Center Groningen) has 24/7/365 occupation of personnel and facilities for acute stroke treatment.

Figure S1. Process map acute stroke pathway.

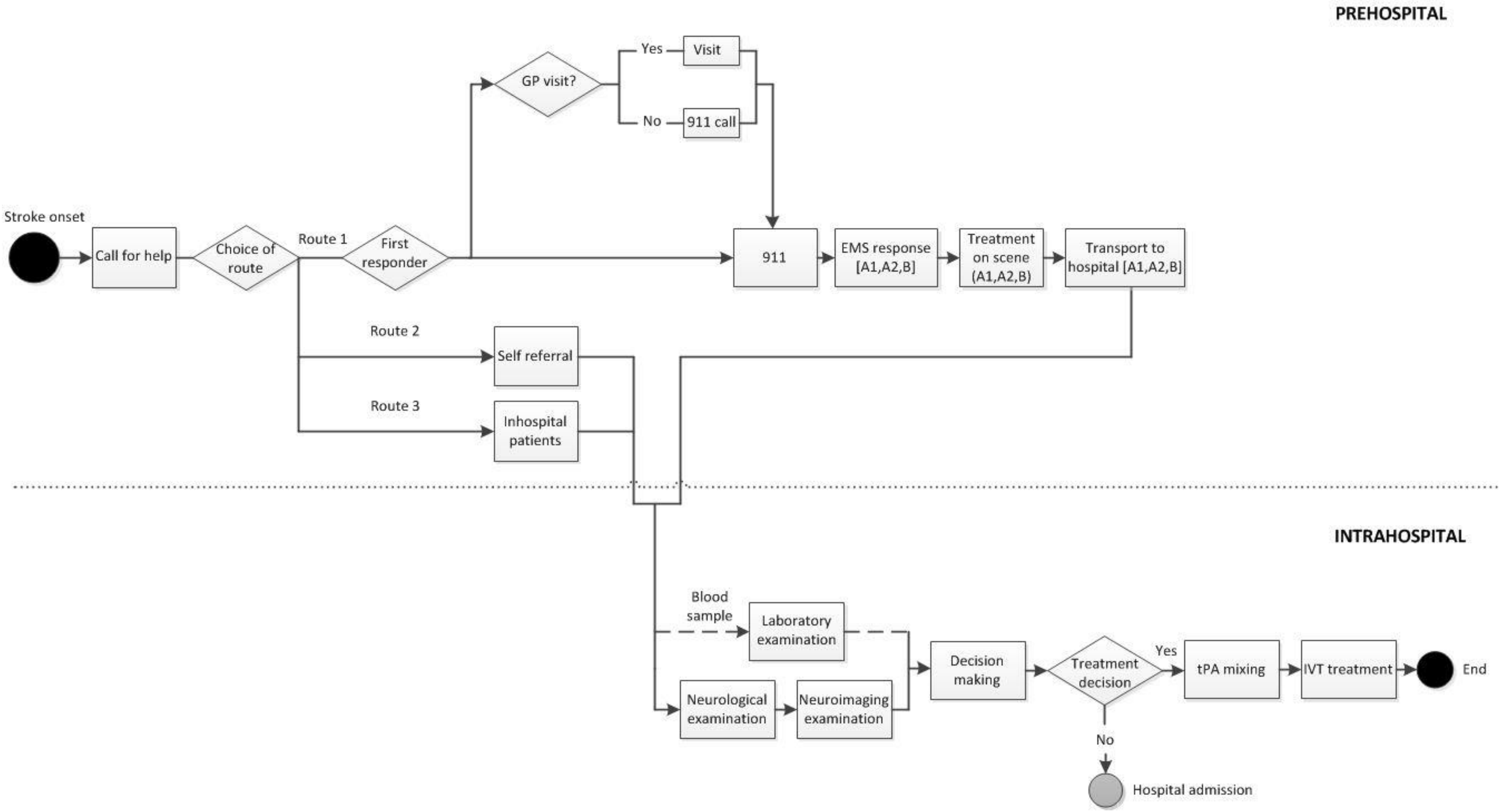

Treatment decision

The efficacy of intravenous tissue plasminogen activator in acute brain infarction is greater the earlier it is administered, and the clinical benefit declines progressively over 4.5 hours after stroke onset.[8] For the simulation model the likelihood of treatment is approximated by a linear function, see Figure S2. We used a linear regression model (Y-axis intercept 97.5; slope -0.33) to approximate the chance of IVT treatment set against the overall process time for all patients arriving $<$ 4.5 hours from the onset of stroke symptoms (i.e. eligible for IVT treatment).

Input parameters

Scenarios studied are modeled by changing input parameters, see Table 2 (main text) for an overview. Choices of input parameter settings reflecting scenarios are shown in Table S1. Alternative settings of input parameters, in terms of modifications to the distributions underlying the baseline scenario, are shown in the most right column of Table S1. References underlying choice of distributions can be found in the main text (Table 2).

Table S1. Distributions and parameters of time delays and diagnostic characteristics.

**Activity duration (minutes)**	**Base line scenario**				**Alternative scenarios**
	**Distribution**	**Distribution parameters**			**Modifications of baseline distributions**
Time from stroke onset to call for help	Continuous empirical	Left bound	Right bound	Frequency (%)	Scenario 1A,B,C (patient responsiveness): Distribution parameters are adjusted by a factor equal to the quotient of the respective median response times reported for best practices, i.e., 30 (1A), 15 (1B), and 0 minutes (1C), and the baseline scenario (41 minutes).
Route 1		0	5	34 (16)	
		5	10	4 (2)	
		10	15	8 (4)	
		15	30	13 (6)	
		30	45	15 (7)	
		45	60	13 (6)	
		60	120	19 (9)	
		120	180	13 (6)	
		180	240	9 (4)	
		240	480	12 (6)	
		480	2880	73 (34)	

Route 2		120	180	1 (2)	
		240	480	1 (2)	
		480	2880	58 (96)	
Route 3		0	5	6 (100)	
Delay first responder					Scenario 5 (expediting response of first responder): Response time is set to zero.
911 call	Uniform	Min (1.00), Max (2.00)			
GP consult by telephone	Uniform	Min (2.00), Max (5.00)			
GP consult by visit	Triangular	Mode (40.00), Min (10.00), Max (30.00)			
Emergency Medical Services					
Response time					Scenario 7A,7B (use of MSU): Response time is set to zero.
A1	Gamma	Alpha (1.36), Beta (6.29)			
A2	Lognormal	Mean (14.21), Standard deviation (6.51)			
B	Beta	Alpha 1 (1.70), Alpha 2 (3.54), a (0.81), b (110.47)			
Time spent on scene					Scenario 6A,B,C (expediting on-scene times): an upper boundary is imposed on distribution outcomes of 15 (6A), 10 (6B) and 0 minutes (6C)
A1	Gamma	Alpha (2.84), Beta (7.42)			Scenario 7B (use of MSU): Transport time is set to zero.
A2	Lognormal	Mean (18.11), Standard deviation (8.39)			
B	Lognormal	Mean (14.25), Standard deviation (8.60)			
Transport time					

A1	Weibull	Alpha (1.93), Beta (19.15)			
A2	Weibull	Alpha (1.43), Beta (16.01)			
B	Beta	Alpha 1 (1.32), Alpha 2 (2.56)			
Time to neurological consultation	Continuous empirical	Left bound	Right bound	Frequency (%)	Scenario 8A,B,C,D (expediting intra hospital processes): an upper boundary is imposed on cumulative distributions reflecting intra hospital processes of 30 (8A), 25 (8B), 20 (8C) and 0 (8D) minutes.
		0	0	93 (76)	
		0	1	4 (3)	
		1	2	7 (6)	
		2	5	6 (5)	
		5	24	12 (10)	
Time to neuroimaging examination	Continuous empirical	Left bound	Right bound	Frequency (%)	
		2	5	28 (23)	
		6	10	54 (44)	
		11	15	13 (11)	
		16	20	10 (8)	
		21	30	8 (7)	
		31	56	8 (7)	
Time to laboratory examination	Erlang	Mean (32.29), Standard deviation (9.26), Location (2.83)			
Decision making	Triangular	Mode (10), Min (5), Max (20)			

IVT mixing	Constant	5		
**Diagnostics**				
Choice of route	Discrete empirical	Value	Frequency (%)	Scenario 2A,B (referral by 911, EMS transport): Distribution parameters, i.e., frequencies have been adjusted such that 60% (2A) and 100% (2B) of patients is referred by 911, and next transported by EMS, implying reduced frequencies for patient self-transport, and GP consults.
1. EMS transport		1	213 (76)	
2. Self-transport		2	60 (21)	
3. Intra-hospital		3	7 (3)	
Choice of first responder if EMS -transport	Discrete empirical	Value	Frequency (%)	
1. 911 call		1	30 (39)	
2. GP consult by phone		2	19 (25)	
3. GP consult by visit		3	27 (36)	
EMS transport, level of urgency	Discrete empirical	Value	%	
911 call				
1. A1		1	95	
2. A2		2	3	
3. B		3	2	
GP consult by telephone				
1. A1		1	88	

2. A2		2	10	
3. B		3	2	
GP consult by visit				
1. A1		1	60	
2. A2		2	33	
3. B		3	7	

Route 1, 2, and 3 indicate patients transported by ambulance, patients arriving at the hospital by self transport, and those patients suffering a stroke while being hospitalized, respectively; GP, general practitioner; A1, A2, B indicate normative values for ambulance arrival within 15, 30, and > 30 minutes from the 911 call until arrival at the location of the patients, respectively; IVT, intravenous thrombolysis; EMS, emergency medical services.

Figure S2. Treatment decision: a patient's chance of being treated given the overall process time.

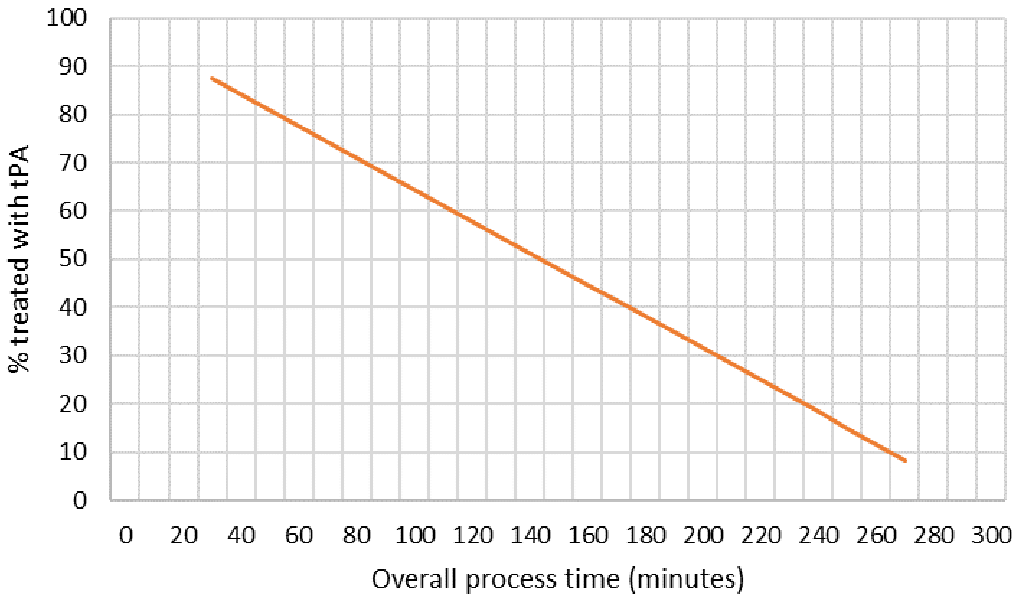

References

1 Law AM. Simulation Modeling and Analysis: McGrawHill: Boston, 5th edition. 2015.

2 Rubinstein RY. Simulation and the Monte Carlo Method: Wiley: Hoboken, 2nd edition 2008.

3 Law AM, Kelton WD. Simulation modeling and analysis. 4th ed.: McGraw-Hill 2007.

4 Monks, T., van der Zee, D. J., Lahr, M., Allen, M., Pearn, K., James, M. A., Buskens, E., Luijckx, G. J. A framework to accelerate simulation studies of hyperacute stroke systems. *Operations Research for Health Care* 2017;15:57-67 doi:https://doi.org/(...)6/j.orhc.2017.09.002.

5 Law AM. ExpertFit Version 8 User's Guide. Tuscon, Arizona: Averill M. Law & Associates 2011.

6 Stahl JE, Furie KL, Gleason S, et al. Stroke: Effect of implementing an evaluation and treatment protocol compliant with NINDS recommendations. *Radiology* 2003;228:659-68 doi:10.1148/radiol.2283021557.

7 Plant Simulation. Siemens PLM 2012. Available at: http://www.plm.automation.siemens.com/en_us/products/tecnomatix/plant_design/plant_simulation.shtml.

8 Lees KR, Bluhmki E, von Kummer R, et al. Time to treatment with intravenous alteplase and outcome in stroke: an updated pooled analysis of ECASS, ATLANTIS, NINDS, and EPITHET trials. *Lancet* 2010;375:1695-703 doi:10.1016/S0140-6736(10)60491-6.
