# Supplementary material for: Optimising acute stroke care organisation: a simulation study to assess the potential to increase intravenous thrombolysis rates and patient gains
Source: BMJ Open. 2020 Jan 20;10(1):e032780. doi: 10.1136/bmjopen-2019-032780 (PMC7045180; doi:10.1136/bmjopen-2019-032780)
Supplement: Author's manuscript [file bmjopen-2019-032780.draft_revisions.pdf]

BMJ Open is committed to open peer review. As part of this commitment we make the peer review history of every article we publish publicly available.

When an article is published we post the peer reviewers' comments and the authors' responses online. We also post the versions of the paper that were used during peer review. These are the versions that the peer review comments apply to.

The versions of the paper that follow are the versions that were submitted during the peer review process. They are not the versions of record or the final published versions. They should not be cited or distributed as the published version of this manuscript.

BMJ Open is an open access journal and the full, final, typeset and author-corrected version of record of the manuscript is available on our site with no access controls, subscription charges or pay-per-view fees (http://bmjopen.bmj.com).

If you have any questions on BMJ Open's open peer review process please email info.bmjopen@bmj.com

BMJ Open

Optimising acute stroke care organisation: a simulation study to assess the potential to increase IVT rates and patient gains

Journal:	*BMJ Open*
Manuscript ID	bmjopen-2019-032780
Article Type:	Research
Date Submitted by the Author:	05-Jul-2019
Complete List of Authors:	Lahr, Maarten ; University of Groningen, University Medical Center Groningen, Health Technology Assessment, Department of Epidemiology<br>van der Zee, Durk-Jouke; Rijksuniversiteit Groningen Faculteit Economie en Bedrijfskunde, Operations<br>Luijckx, Gert-Jan; University of Groningen, University Medical Center Groningen, Neurology<br>Buskens, Erik; University of Groningen, University Medical Center Groningen, Epidemiology; University of Groningen, Faculty of Economics and Business, Deparment of Operations
Keywords:	Stroke < NEUROLOGY, Organisation of health services < HEALTH SERVICES ADMINISTRATION & MANAGEMENT, EPIDEMIOLOGY

SCHOLARONE™
Manuscripts

Optimising acute stroke care organisation: a simulation study to assess the potential to increase IVT rates and patient gains

Maarten M.H. Lahr[1] Ph.D., Durk-Jouke van der Zee[2] Ph.D., Gert-Jan Luijckx[3] M.D., Ph.D., Erik Buskens[1,2] M.D., Ph.D.

[1] *Health Technology Assessment, Department of Epidemiology, University of Groningen, University Medical Centre Groningen, Groningen, The Netherlands*
[2] *Department of Operations, Faculty of Economics & Business, University of Groningen, Groningen, The Netherlands*
[3] *Department of Neurology, University of Groningen, University Medical Centre Groningen, Groningen, The Netherlands*

For peer review only

Word count: 3602
Number of tables: 3
Number of figures: 2

Corresponding Author:
Maarten M.H. Lahr
University Medical Centre Groningen, Department of Epidemiology
P.O. Box 30001. 9700 RB Groningen, The Netherlands
Telephone: + 31 50 361 43 86
E-mail: m.m.h.lahr@umcg.nl

Abstract

Objectives To assess potential increases in Intravenous Thrombolysis (IVT) rates given particular interventions in the stroke care pathway.

Design Simulation modelling was used to compare the performance of the current pathway, best practices based on literature review, and an optimised model. The interventions investigated included efforts aimed at patient response and mode of referral, prehospital triage, and intra-hospital delays.

Setting Four hospitals located in the north of the Netherlands as part of a centralised organisational model.

Participants A total of 280 ischaemic stroke patients were ascertained between February to August 2010.

Primary and secondary outcome measures The primary outcome measure was thrombolysis utilisation. Secondary measures were onset-treatment time (OTT) and the proportion of patients with excellent functional outcome (mRS 0-1) at 90 days.

Results
Of 280 patients with ischaemic stroke, 125 (44.6%) arrived at the hospital within 4.5 hours, and 61 (21.8%) received IVT. The largest improvements in IVT treatment rates, OTT, and the proportion of patients with mRS scores of 0-1 can be expected when patient response is limited to 15 minutes (IVT rate +5.8%; OTT -6 minutes; excellent mRS scores +0.2%), door-to-needle time to 20 minutes (IVT rate +4.8%; OTT -28 minutes; excellent mRS scores +3.2%), and 911 calls are increased to 60% (IVT rate +2.9%; OTT -2 minutes; excellent mRS scores +0.2%). The combined implementation of all potential best practices could increase IVT rates by 19.7% and reduce OTT by 56 minutes.

Conclusions
Improving IVT rates to well above 30% appears possible if all known best practices are implemented.

Key words: acute stroke, thrombolysis, organisational model, simulation modelling, quality improvement.

Strengths and limitations of the study

- The proposed simulation modelling study uses real-world patient level data rather than those derived from the literature.
- The proposed methodology can serve as a template for other regions and health care systems to guide optimisation initiatives.
- The modelling approach has a comprehensive scope on acute care delivery, as it includes both prehospital and intrahospital data.
- Model results are estimations which have to be tested in clinical practice.
- Input parameters for model building contain estimations of time delays and diagnostic procedures that may have changed over time.

For peer review only

INTRODUCTION

Intravenous thrombolysis (IVT) is an effective therapy for acute ischaemic stroke up to 4.5 hours after the onset of symptoms.[1, 2] This therapy is substantially underused, however, with 8-10% of all stroke patients worldwide currently receiving IVT.[3, 4] In contrast, treatment rates of up to 35% have been achieved in optimised settings.[5, 6] The organisation of stroke care is an important factor in realising timely hospital arrival and treatment.[7, 8] The centralisation of care at designated stroke centres has been demonstrated to increase the proportion of patients arriving at the hospital in time for acute treatment, in addition to increasing the use of evidence-based clinical interventions and reducing mortality.[9-11] Given the substantial decrease in the benefit of treatment with increasing time delays, further efforts to expedite hospital arrival and subsequent treatment remain of crucial importance.[12, 13]

Various studies have investigated factors associated with efficiency in each part of the acute stroke pathway, ranging from patient responsiveness to pathway set-up. Although it has been generally established that delay on the part of patients and/or bystanders is a primary factor in delaying hospital arrival,[14] interventions aimed at improving optimal response by calling 911 immediately have exhibited varying success, and many lack sustained implementation[15] (as is the case in other domains of medicine as well).[16] Ambulance transportation to hospitals that offer acute treatment is associated with shorter onset-to-door times, reductions in intra-hospital delays, and increases in treatment rates.[15, 17, 18] The provision of acute treatment by a Mobile Stroke Unit (MSU) before hospital arrival has been identified as a promising method for reducing time to IVT. The beneficial effects of such treatment on patient outcomes and cost-effectiveness have yet to be demonstrated.[19] Another widely studied topic concerns reducing the time between hospital arrival and treatment (door-to-needle time, or DTN), with reported DTN times as low as 20 minutes.[20]

The aforementioned studies have demonstrated clear benefits in terms of time saved. They nevertheless lack a broader overview of pathway set-up and its performance. Instead of addressing the solution space as a whole, they target isolated elements of the stroke pathway. They are therefore likely to overlook potential solutions lying outside the scope of the particular study but impacting relevant clinical outcomes. The lack of a broader overview is in part due to the predominant use of randomized controlled trials (RCT) as the main research vehicle. Given the effort and resources involved in their set-up, and the inherent limitation in flexibly incorporating various potential solutions RCTs predominantly address separate and singular elements of pathway performance. This makes them less suitable for investigating complex care systems (e.g., acute stroke treatment). In particular, timely hospital arrival and the treatment of acute stroke patients relies on a series of intertwined activities concerning patient diagnostics and transportation, which are provided by staff with a variety of skills and expertise, and which are therefore supported by various resources.

One potential alternative methodology is simulation modelling. Proceeding from a detailed description of both pre-hospital and intra-hospital time delays and diagnostics, an accurate representation of pathway performance can be developed *in silico*, including the validation of IVT rates, time to treatment, patient outcomes (as measured by the modified Rankin Scale or mRS), and other clinically relevant outcome measures.[21, 22] This approach would allow the examination of all time delays and diagnostic steps, from the onset of symptoms to treatment in the hospital, thereby providing clinicians and policymakers with an accurate overview of obstacles currently existing within care pathways. In addition, scenarios for hypothetical approaches to improvement based on clinical guidelines, literature observations, and/or expert opinion can be incorporated and studied for their cumulative effects on relevant clinical outcome measures.[23, 24] The tradeoff obviously is that although seemingly viable solutions will be identified, there is no ‘proof of the pudding’ yet. Robust ‘in

For peer review only - http://bmjopen.bmj.com/site/about/guidelines.xhtml

silico' experiments will take away huge amounts of decision uncertainty, yet, will not yield 2-decimal 95% confidence intervals on factually observed effects or costs.

Nevertheless, we remain confident that we will be able to guide policymakers and set out on this simulation-modelling study to (1) estimate the cumulative potential for improving IVT utilisation by implementing best practices on the organisation of the stroke pathway and, subsequently, (2) to explore areas in which further improvement is needed in order to achieve a fully optimised setting, in addition to identifying obstacles to such optimisation.

METHODS

This article is based on a six-month, prospective, multi-centre study performed in a centralised organisational stroke-care setting in the north of the Netherlands from February through July 2010.[9] Patient-level data were collected on time delays and diagnostics, thereby providing detailed insight into patient flow and potential obstacles in both the pre-hospital and intra-hospital pathways (Table 1). A schematic overview of patient flow and the steps included in the analyses is presented in Figure 1.

Table 1. Descriptive statistics of activity durations and diagnostics

Number of patients	280
Age in years (SD)	70 (14)
Male (%)	156 (56)
**Patient responsiveness**	
Time from symptom onset to call for help, valid cases (%)	152 (54)
Median, minutes (IQR)	41 (5-130)
Mode of referral (%)	
General practitioner	129 (46)
911	84 (30)
Self-referral	60 (21)
In-hospital patients	7 (3)
**Pathway set-up**	
Transported by EMS (%)	213 (76)
Response time, median (min)	9 (7-12)
On scene time, median (min)	20 (15-25)
Transportation time, median (min)	17 (9-22)
Median time from hospital arrival to neurological examination, min (IQR)	2 (0-0)
Median time from hospital arrival to CT examination min (IQR)	12 (6-15)
Median time from hospital arrival to laboratory examination, min (IQR)	32 (27-37)
Median door to IVT time, min (IQR)	35 (25-45)

SD indicates standard deviation; IQR, Interquartile Range; EMS, Emergency Medical Services; CT, Computed Tomography; IVT, Intravenous Thrombolysis.

For peer review only

Setting and participants

The centralised organisational setting consisted of four hospitals in the northern region of the Netherlands, with IVT being administered only in the University Medical Centre Groningen (UMCG). Together with the other three community hospitals, general practitioner (GP) offices, and emergency medical services (EMS), arrangements were made to transport presumed stroke victims immediately to the UMCG, thus bypassing community hospitals that might have been located closer to the patient's location. The international protocol for IVT (adjusted ECASS III[25]) and the regional protocol for pre-hospital management were followed. The region addressed in this study comprises approximately 580,000 inhabitants, with a population density of 250 inhabitants per square kilometer. The study population consisted of ischaemic stroke patients admitted to all four hospitals between February and August 2010. Case ascertainment was confirmed by the final hospital discharge diagnosis of ischaemic stroke, thereby excluding stroke mimics.

The data collected included information on time delays and diagnostics along the entire stroke chain. Time delays included the time from symptom onset to call for help, delay at first response (GP or EMS services), EMS transport times, and intra-hospital diagnostics, which included time from hospital arrival to neurological examination, Computed Tomography (CT) scan, laboratory testing, and IVT.

Simulation model

The current study was performed using a previously validated simulation model. This model was populated with patient-level data from a previous observational study.[9] The model was validated by comparing IVT treatment percentages and OTT to those reported in the observational study. The next step consisted of developing scenarios in which alternative pathway set-ups, associated time delays, and diagnostics were imputed based on literature observations, clinical guidelines, and expert opinion. Using the simulation model, hypothetical patients were passed through the system, estimating the impact of intervening at various points in the acute stroke pathway. Interventions were modelled by changing the underlying statistical distributions to redistribute patients across time delays and diagnostics.[26]

Scenarios

We used simulation modelling to investigate the effects of changing pathway set-ups, based on three models: (1) a baseline model of acute stroke care; (2) a model reflecting best practices, based on a review of the current literature, clinical guidelines, and expert opinion; and (3) an optimised model. Interventions were selected according to the obstacles identified in the current centralised organisational model. Obstacles within the study setting included delayed emergency response by patients following symptom onset, mode of referral (GP or 911), time spent on the scene by ambulance personnel, and intra-hospital delays. The simulation model was used to perform hypothetical interventions in the pathway to calculate clinically relevant outcomes.[24] The outcomes were compared to the baseline performance of the current system to estimate the potential for improvement, to optimise system performance, and to identify obstacles that have yet to be overcome.

Baseline

The baseline model describes the performance of the centralised organisational model for acute stroke care, as described in the previous observational study.[9] A description of time delays and diagnostics along each step of the pathway is provided in Table 1.

For peer review only - http://bmjopen.bmj.com/site/about/guidelines.xhtml

Best practice

The scenarios that were investigated are described in Table 2.

Patient responsiveness: We estimated the relative impact of reducing the time between stroke onset and call for help by patients, their families, and/or bystanders by adjusting the distribution of their response times by a factor equal to the quotient of the respective median response times reported for best practices (15-30 minutes)[27, 28] and the baseline scenario (41 minutes).

Mode of referral: We modelled a scenario in which patients, their families, and/or bystanders predominantly (60%) chose 911 as the mode of entry.[29, 30]

Time spent on the scene by ambulance personnel: In this scenario, we modelled the time spent on the scene by ambulance personnel following a 911 call by imposing an upper boundary on on-scene delay. Based on the guidelines of the American Stroke Association, the time spent on the scene should be no more than 15 minutes.[31] We also modelled the implementation of a "scoop and run protocol," which includes prompt transport (≤ 10 minutes) with initial management efforts, while postponing elaborate triage.

Pre-hospital treatment by MSU: In this scenario, we modelled pre-hospital IVT provided by a MSU by adjusting only the transport time of the ambulance. Assuming that the MSU would be stationed at the hospital, we considered only the transportation time from the hospital to the patient scene.

For peer review only

Table 2. Overview of scenarios.

**Patient responsiveness**	**Baseline**	**Best practice**	**Study**	**Optimized**
Patient delay, median (min)	41	30	Salisbury et al. (1998)	0
		15	Carrol et al. (2004)	
Mode of referral 911 (%)	30	60	Barsan et al. (1994)<br>Chen et al. (2013)	100
**Pathway set-up**				
Transportation EMS				
Response time, median (min)	9	-	-	0
On scene time, median (min)	20	15	Jauch et al. (2013), Acker et al. (2007)	0
		10	Atkins et al. (2009)	
Transportation time, median (min)	17	-		0
Prehospital IVT by MSU	-	Transportation time = 0	-	Response time = 0<br>Transportation time = 0
Door-to-IVT time, median (min)	35	30	Zinkstok et al. (2016)	0
		25	Meretoja et al. (2013)	
		20	Meretoja et al. (2012)	

EMS indicates emergency medical services; IVT, intravenous thrombolysis; MSU, Mobile Stroke Unit.

For peer review only

Intra-hospital processes: Intra-hospital delays comprise all activities performed between arrival at the hospital until the start of IVT (door-to-needle time; see Figure 1). Based on the guidelines of the American Stroke Association, door-to-needle time should be no more than 60 minutes.[32] Evidence from clinical practice suggests that DTN times within a range of 20 to 30 minutes are attainable.[20, 33, 34]

Combined best-practice scenarios

Three scenarios were performed in which best practices were combined for patient responsiveness and pathway set-up. Patient responsiveness was modelled by combining patient response with the mode of referral following stroke onset. Pathway set-up was modelled by combining pre-hospital and intra-hospital best practices. A third scenario combines best practices for patient responsiveness and pathway set-up.

Optimised scenarios

Additional scenarios were defined to interpret findings on the effects of the implementation of best practices (or combinations thereof) by generating upper boundaries to pathway performance, thereby building on unrealistically optimistic assumptions (see the last column in Table 2). For the optimised scenarios, we extrapolated best practices by setting the parameter for associated time delays to 0 or by setting the diagnostic quality parameter to 100%.

Outcome measures

The primary outcome measure was the proportion of patients treated with IVT. Secondary outcome measures were the total process time (onset-to-treatment time), IVT within various time intervals (0-90, 91-180, and 181-270 minutes), the proportion of patients with favorable outcomes at 90 days (modified Rankin Scale 0-1), and additional healthy life days (calculated using OTT estimates).[12, 35]

Analysis

For each of the scenarios described above, we calculated new hypothetical IVT treatment rates and secondary outcome measures, based on the number of patients arriving in time for acute treatment at the hospital (i.e., within 4 hours after symptom onset), the number treated with IVT, and the time to treatment.

Informed consent

Informed consent was obtained from all subjects participating in the prospective study and extended for the current simulation study.

Patient and public involvement

We did not directly involve patients in the design or execution of the study. However, the data used for the analyses was based on patient involvement as it was prospectively collected.

RESULTS

This study reflects the experiences of 280 ischaemic stroke patients referred to the UMCG and three community hospitals, as part of a centralised organisational model. Baseline and demographic characteristics are described in Table 1. In all, 125 (44.6%) patients arrived at the hospital within 4.5 hours, 61 (21.8%) received IVT, and the median OTT was 127 minutes. Of the patients receiving IVT, 17.0% were treated within 90 minutes of symptom onset. Patient delay, intra-hospital delays, and mode of referral (GP or 911) were identified as the greatest obstacles to receiving IVT (Table 1).

For peer review only

Simulation experiments

The results of the simulation experiments are presented in Table 3. The improvements achieved by best practices in closing the gap between results of the baseline model and optimised settings are summarised in Figure 2.

For peer review only

Table 3. Re-configuration centralised model: results simulation experiments.

	IVT rate (95% CI)	OTT minutes (95% CI)	IVT 0-1.5 hours	IVT 1.5-3.0 hours	IVT 3.0-4.5 hours	mRS 0-1†	Extra healthy days‡
**Scenario**							
0.Current practice	21.8% (20.9% - 22.6%)	129 (127 – 130)	17.0%	70.9%	12.1%	12.5%	
**Patient responsiveness**							
1.Patient delay							
A. Reduced to 30 minutes	23.7% (22.9% - 24.6%)	127 (125 – 129)	16.9%	72.2%	10.9%	12.6%	2.9
B. Reduced to 15 minutes	27.6% (26.7% - 28.4%)	122 (121 – 124)	16.5%	76.7%	6.8%	12.7%	11.3
C. Reduced to 0 minutes	64.0% (63.0% - 64.9%)	92 (91 – 92)	45.7%	54.1%	0.2%	16.2%	66.9
2. Mode of referral 911							
A. Increased to 60%	24.6% (23.8% - 25.5%)	127 (125 – 128)	18.8%	70.0%	11.2%	12.8%	3.9
B. Increased to 100%	28.4% (27.5% - 29.3%)	124 (123 – 126)	18.3%	71.4%	10.3%	12.7%	7.8
3. Combined best practices patient responsiveness (1B+2A)	31.0% (30.1% - 31.9%)	120 (118 – 121)	19.2%	74.4%	6.4%	13.0%	16.3
4. Optimised patient responsiveness (1C + 2B)	64.3% (63.3% - 65.2%)	98 (97 – 98)	36.2%	63.7%	0.1%	15.1%	55.7
**Pathway set-up**							
5. EMS response time reduced to 0 minutes	23.3% (22.4% - 24.1%)	121 (119 - 122)	21.8%	68.9%	9.3%	13.2%	14.4
6. On scene time ambulance personnel							
A. Reduced to 15 minutes	22.8% (21.9% - 23.6%)	124 (122 – 126)	19.4%	69.9%	10.7%	12.8%	8.6
B. Reduced to 10 minutes	23.3% (22.4% - 24.1%)	121 (120 – 123)	21.7%	68.5%	9.8%	13.1%	13.4
C. Reduced to 0 minutes	24.7% (23.8% - 25.5%)	114 (112 – 116)	31.4%	60.4%	8.2%	14.3%	26.3
7. Prehospital IVT by MSU							
A. Transportation time reduced to 0 minutes	23.2% (22.4% - 24.0%)	121 (120 – 123)	22.8%	67.4%	9.8%	13.2%	13.1
B. All ambulance delays reduced to 0 minutes	25.6% (24.7% – 26.4%)	109 (107 – 110)	38.6%	54.3%	7.0%	15.1%	35.8
8. Door to IVT times							
A. Reduced to 30 minutes	25.2% (24.3% - 26.0%)	110 (108 – 111)	34.3%	57.7%	8.0%	14.6%	34.4
B. Reduced to 25 minutes	25.9% (25.0% - 26.8%)	106 (104 – 107)	39.3%	53.2%	7.5%	15.2%	41.5
C. Reduced to 20 minutes	26.6% (25.7% - 27.4%)	101 (99 – 103)	44.2%	48.8%	7.0%	15.7%	49.8
D. Reduced to 0 minutes	29.8% (28.9% - 30.7%)	83 (81 – 85)	62.9%	32.1%	5.0%	17.9%	81.9
9. Combined best practices pathway set-up (6B+7A+8C)	30.8% (29.9% - 31.7%)	80 (79 – 82)	68.9%	26.3%	4.8%	18.6%	87.3
10. Optimised pathway set-up (5 + 6C + 7B + 8D)	38.5% (37.6% - 39.5%)	39 (37 – 40)	86.0%	11.3%	2.7%	20.6%	161.7
**Patient responsiveness and pathway set-up**							
11. Combined best practices patient responsiveness and	41.5% (40.5% - 42.4%)	73 (72 – 74)	77.4%	20.5%	2.2%	19.6%	99.9

For peer review only

pathway set-up (3+9)							
12. Optimised patient responsiveness and pathway set-up (5+10)	97.7% (97.4% – 98.0%)	0 (0 – 0)	100.0%	0.0%	0.0%	22.2%	231.6

IVT indicates intravenous thrombolysis; CI, confidence interval; OTT, onset-treatment-time; tPA indicates tissue plasminogen activator; mRS, modified rankin scale; MSU, mobile stroke unit.

† Indicates the proportion of patients with good outcome (mRS 0-1) ascribed to treatment with thrombolysis.[33]

‡ Indicated the number of additional days in healthy life by minute reduction in OTT.[12]

For peer review only

Patient responsiveness: If patients had contacted emergency services sooner (i.e., within 30 and 15 minutes), up to 27.6% (CI 26.7% – 28.4%) of the total population would have been treated with IVT (Table 3, Scenarios 1A and 1B), and the OTT would have been reduced to 122 minutes (CI 121 – 124).[27, 28]

Assuming a patient delay of 0 minutes (Table 3, Scenario 1C), 64.0% of the total population would have been treated with IVT, and the OTT would have been reduced to 92 minutes (CI 91 – 92).

Mode of referral: Assuming 60% of all patients contacting 911 immediately following stroke onset (Table 3, Scenario 2A) increased the IVT rate to 24.6% (CI 23.8% – 25.5%) and reduced the OTT to 127 minutes (CI 125 – 128).[29]

If all patients (100%) had called 911 (Table 3, Scenario 2B), the IVT rates would have increased further to 28.4% (CI 27.5% – 29.3%), and the OTT would have been reduced to 124 minutes (CI 123 – 126).

Time spent on the scene by ambulance personnel: Shortening the time spent on the scene to 15 and 10 minutes (Table 3, Scenarios 6A and 6B),[36] increased the IVT treatment rate up to 23.3% (CI 22.4% – 24.1%) and reduced the OTT to 121 minutes (CI 120 – 123).[37, 38]

Reducing time on the scene to 0 minutes resulted in a projected IVT rate of 24.7% (CI 23.8% – 25.5%) (Table 3, Scenario 6C) and a decrease in the OTT to 114 minutes (CI 112 – 116).

Pre-hospital treatment by Mobile Stroke Unit (MSU): In this set-up, 23.2% (CI 22.4% – 24.0%) of patients would have been treated with IVT (Table 3, Scenario 7A) and OTT would have been reduced to 121 minutes (CI 120 – 123).

The elimination of both the response time and transportation time of the MSU (Table 3, Scenario 7B) resulted in a projected 25.6% (CI 24.7% - 26.4%) of patients that could have received IVT and a reduction of the OTT to 109 minutes (CI 107 – 110).

Intra-hospital processes: If the DTN time had been shortened to a maximum of 30 and 20 minutes (Table 3, Scenarios 8A-C), up to 26.6% (CI 25.7% - 27.4%) of the total population would have been treated with IVT, and the OTT would have been reduced to 101 minutes (CI 99 – 103).

If the DTN had been reduced to 0 minutes (Table 3, Scenario 8D), 29.8% (CI 28.9 – 30.7%) of all patients would have been treated with IVT, and the OTT would have been reduced to 83 minutes (CI 81 – 85).

Combined best practice scenarios

Combining best practices for patient responsiveness and pathway set-up (Table 3, scenario's 3,9,11) resulted in up to 41.5% (CI 40.5% - 42.4%) of all patients being treated with IVT and reduced the OTT to 73 minutes (CI 72 – 74).

Optimised scenarios

Assuming optimised patient responsiveness (i.e. all patients calling 911 immediately following stroke onset; Table 3, Scenario 4) resulted in 64.3% of the total population (CI 63.3% – 65.2%) being treated with IVT and reduced the OTT to 98 minutes (CI 97 – 98). The optimisation of pathway set-up (Table 3, Scenario 10) resulted in 38.5% (CI 37.6% – 39.5%) of all patients receiving IVT and reduced the OTT to 39 minutes (CI 37 – 40). The combination of all optimised scenarios (Table 3, Scenario 12) resulted in a cumulative total of 97.7% (CI 97.4% - 98.0%) of all patients being treated with IVT and reduced the OTT to 0 minutes.

For peer review only

DISCUSSION

This study demonstrates that IVT treatment rates above 30% may be possible if best practices were concurrently implemented within our setting. We modelled several scenarios to generate insight into the potential for quality improvements in our acute stroke chain of care. Although improvements in patient responsiveness would yield the largest potential gains within our pathway, even modest changes in this regard are likely to be challenging and costly to achieve.[16] In contrast, improvements in other areas (e.g., intra-hospital delays and time spent by ambulance personnel at the patient's location) might be easier to achieve and would still lead to clinically relevant increases in IVT rates. As indicated in previous studies, even small reductions in time to treatment with IVT are associated with considerable increases in the length of healthy life, and they may require only relatively simple organisational changes involving minimal effort at little or no cost.[12]

Despite the seeming absence of sound clinical proof the results of our study clearly provide rather precise numerical indications of the potential for improvement within the acute stroke services. We assert that such information is very useful as a guide for prioritising interventions and for estimating their potential impact on the effectiveness of the services. A simulation-based approach, as presented in this paper, can be instrumental in facilitating a broad overview of the set-up and performance of stroke pathways. This could provide clinicians and policymakers with speedy answers – at little effort or cost – concerning how new or widely advocated practices could be used to improve their pathways, thus allowing them to direct investments to the interventions that matter most. It thus has the potential to replace RCT studies or serve as a precursor to a focused RCT, which could be scoped as the net result of a simulation approach.

In our study, we observed the greatest effects on IVT rates, time to treatment, and patient outcomes after improving the responsiveness of patients and/or bystanders by reducing the time from symptom onset to the call for help, thereby expediting intra-hospital care services and by increasing the number of 911 calls made by patients or bystanders. In contrast, a scenario in which pre-hospital transportation delays were reduced by the implementation of a MSU resulted in only moderate effects. Combining all of the best-practice scenarios resulted in a maximum of 41.5% of patients being treated with IVT. This is substantially higher than current benchmark figures on clinical practice, which suggest a maximum IVT rate of around 35%.[6, 39]

Proceeding from a scenario in which best practices have been implemented, remaining challenges include realising further decreases in time delays in both the pre-hospital and intra-hospital phases. The feasibility of such initiatives in clinical practice might be limited in the short term, however, given the current lack of evidence concerning solutions for further expediting care and logistics services at reasonable costs. For example, the goal of reducing time spent on the scene by ambulance personnel to less than 10 minutes or reducing door-to-needle time to less than 20 minutes would probably be unrealistic, given the need to handle and observe the patient, to complete diagnostic tests, and to interpret findings. Although further improvements in the proportion of patients calling 911 directly following symptom onset could potentially result in further increases in IVT rates, they would also necessitate large-scale and repetitive publicity campaigns comparable to those launched to raise public awareness on stroke symptoms and how to act.

The organisational model for acute stroke care delivery is currently receiving a great deal of attention in the Netherlands, as well as beyond.[40, 41] The emergence of endovascular treatment (EVT) for patients facing large-vessel occlusions has opened up a whole new dimension in terms of acute stroke pathway set-up and patient logistics. Following IVT, eligible patients must now undergo additional diagnostic evaluation (e.g., CT angiography and perfusion CT), followed by such EVT treatment modalities as groin punctures and initial

For peer review only

attempt at clot retrieval with the device up to the angioseal following successful recanalisation. In addition, within the current "drip-and-ship" treatment paradigm, eligible patients may initially be admitted to community hospitals before being transferred to comprehensive stroke centres with EVT capacity, thereby further increasing the number of logistical steps. Given the time-sensitive nature of acute stroke interventions, this extension of the pathway necessitates the re-organisation of acute stroke care within regions and settings. In this respect, simulation modelling could facilitate insight into the complex interplay of separate elements of the pathway.

Our study is subject to several limitations. First, our simulation models did not consider the response of GPs when contacted as first responders. Although this has been signaled as an issue for delays in hospital arrival for patients,[42] no studies on best practices were identified in the literature. Second, because the costs and cost-effectiveness associated with pathway improvements in our setting were not estimated in our model, it was not possible to control for them. Third, the results of our findings might not be generalisable to other settings, due to the unique position of the UMCG, which serves as a stroke centre in a centralised organisational model deployed within its region. As noted in a previous publication, however, the generic modelling approach adopted in this study could be extended to include a description of a decentralised organisational model, which receives IVT candidates within its own catchment areas.[23] Also, because patients were enrolled in the observational study back in 2010 these results will not fully reflect current practice. However, review of internal databases show that IVT treatment percentages have remained largely stable over the last years, fluctuating around 25%. Future activities should be aimed at extending the simulation-based approach to include the drip-and-ship model currently employed in acute stroke treatment (e.g., EVT).

CONCLUSIONS

The results of this study indicate that the cumulative effects of implementing best practices within the organisation of stroke care can be expected to noticeably exceed current benchmarks for treatment rates. Remaining obstacles might be difficult to overcome given the limited availability of feasible interventions to further expedite care and services at acceptable costs. A broader overview facilitated by simulation is suggested as instrumental in supporting decision-makers and clinicians in their efforts to evaluate the set-up and performance of acute stroke pathways.

Acknowledgements None

Contributors ML, DJZ, GJL and EB designed the study. ML performed the statistical analyses and drafted the manuscript. DJZ, GJL and EB critically revised the manuscript for intellectual content and approved the final version of the manuscript for publication.

Funding Not applicable

Competing interest statement The authors declare that there is no conflict of interest.

Figure 1. Acute stroke pathway: description of activities.
Figure 2. Summary of best practices and relative gap (%) between baseline model and optimized settings. This figure depicts the total relative gap in IVT rates between the baseline model and the optimized scenarios (i.e. time delays set at zero and diagnostics at 100%). The colored bars represent the relative gains in IVT rates, per best practice (A-D) in each of the improvement domains. Also the contribution of combined best practices are considered.

For peer review only

REFERENCES

1 . Tissue plasminogen activator for acute ischemic stroke. The National Institute of Neurological Disorders and Stroke rt-PA Stroke Study Group. *N Engl J Med* 1995;333:1581-7 doi:10.1056/NEJM199512143332401.

2 Hacke W, Kaste M, Bluhmki E, et al. Thrombolysis with alteplase 3 to 4.5 hours after acute ischemic stroke. *N Engl J Med* 2008;359:1317-29 doi:10.1056/NEJMoa0804656.

3 Bauer A, Limburg M, Visser MC. Variation in clinical practice of intravenous thrombolysis in stroke in the Netherlands. *Cerebrovasc Dis Extra* 2013;3:74-7 doi:10.1159/000350707; 10.1159/000350707.

4 Adeoye O, Hornung R, Khatri P, et al. Recombinant tissue-type plasminogen activator use for ischemic stroke in the United States: a doubling of treatment rates over the course of 5 years. *Stroke* 2011;42:1952-5 doi:10.1161/STROKEAHA.110.612358.

5 Waite K, Silver F, Jaigobin C, et al. Telestroke: a multi-site, emergency-based telemedicine service in Ontario. *J Telemed Telecare* 2006;12:141-5 doi:10.1258/135763306776738611.

6 Stolz E, Hamann GF, Kaps M, et al. Regional differences in acute stroke admission and thrombolysis rates in the German federal state of Hesse. *Dtsch Arztebl Int* 2011;108:607-11 doi:10.3238/arztebl.2011.0607 [doi].

7 Bekelis K, Marth NJ, Wong K, et al. Primary Stroke Center Hospitalization for Elderly Patients With Stroke: Implications for Case Fatality and Travel Times. *JAMA Intern Med* 2016;176:1361-8 doi:10.1001/jamainternmed.2016.3919 [doi].

8 Willeit J, Geley T, Schoch J, et al. Thrombolysis and clinical outcome in patients with stroke after implementation of the Tyrol Stroke Pathway: a retrospective observational study. *Lancet Neurol* 2015;14:48-56 doi:10.1016/S1474-4422(14)70286-8 [doi].

9 Lahr MM, Luijckx GJ, Vroomen PC, et al. Proportion of patients treated with thrombolysis in a centralized versus a decentralized acute stroke care setting. *Stroke* 2012;43:1336-40 doi:10.1161/STROKEAHA.111.641795.

10 Ramsay AI, Morris S, Hoffman A, et al. Effects of Centralizing Acute Stroke Services on Stroke Care Provision in Two Large Metropolitan Areas in England. *Stroke* 2015;46:2244-51 doi:10.1161/STROKEAHA.115.009723 [doi].

11 Morris S, Hunter RM, Ramsay AI, et al. Impact of centralising acute stroke services in English metropolitan areas on mortality and length of hospital stay: difference-in-differences analysis. *BMJ* 2014;349:g4757 doi:10.1136/bmj.g4757 [doi].

12 Meretoja A, Keshtkaran M, Saver JL, et al. Stroke thrombolysis: save a minute, save a day. *Stroke* 2014;45:1053-8 doi:10.1161/STROKEAHA.113.002910 [doi].

13 Tsivgoulis G, Katsanos AH, Kadlecova P, et al. Intravenous thrombolysis for ischemic stroke in the golden hour: propensity-matched analysis from the SITS-EAST registry. *J Neurol* 2017;264:912-20 doi:10.1007/s00415-017-8461-8 [doi].

For peer review only

14 Kleindorfer D, Khoury J, Broderick JP, et al. Temporal trends in public awareness of stroke: warning signs, risk factors, and treatment. *Stroke* 2009;40:2502-6 doi:10.1161/STROKEAHA.109.551861 [doi].

15 Fassbender K, Balucani C, Walter S, et al. Streamlining of prehospital stroke management: the golden hour. *Lancet Neurol* 2013;12:585-96 doi:10.1016/S1474-4422(13)70100-5; 10.1016/S1474-4422(13)70100-5.

16 Wakefield MA, Loken B, Hornik RC. Use of mass media campaigns to change health behaviour. *Lancet* 2010;376:1261-71 doi:10.1016/S0140-6736(10)60809-4.

17 Evenson KR, Foraker RE, Morris DL, et al. A comprehensive review of prehospital and in-hospital delay times in acute stroke care. *Int J Stroke* 2009;4:187-99 doi:10.1111/j.1747-4949.2009.00276.x.

18 Rose KM, Rosamond WD, Huston SL, et al. Predictors of time from hospital arrival to initial brain-imaging among suspected stroke patients: the North Carolina Collaborative Stroke Registry. *Stroke* 2008;39:3262-7 doi:10.1161/STROKEAHA.108.524686; 10.1161/STROKEAHA.108.524686.

19 Fassbender K, Grotta JC, Walter S, et al. Mobile stroke units for prehospital thrombolysis, triage, and beyond: benefits and challenges. *Lancet Neurol* 2017;16:227-37 doi:S1474-4422(17)30008-X [pii].

20 Meretoja A, Strbian D, Mustanoja S, et al. Reducing in-hospital delay to 20 minutes in stroke thrombolysis. *Neurology* 2012;79(4):306-13 doi:10.1212/WNL.0b013e31825d6011.

21 Lahr MM, van der Zee DJ, Vroomen PC, et al. Thrombolysis in acute ischemic stroke: a simulation study to improve pre- and in-hospital delays in community hospitals. *PLoS One* 2013;8:e79049 doi:10.1371/journal.pone.0079049 [doi].

22 Monks T, Pitt M, Stein K, et al. Maximizing the Population Benefit From Thrombolysis in Acute Ischemic Stroke: A Modeling Study of In-Hospital Delays. *Stroke* 2012;43(10):2706-11 doi:10.1161/STROKEAHA.112.663187.

23 Lahr MM, van der Zee DJ, Luijckx GJ, et al. Centralising and optimising decentralised stroke care systems: a simulation study on short-term costs and effects. *BMC Med Res Methodol* 2017;17:5,016-0275-3 doi:10.1186/s12874-016-0275-3 [doi].

24 Lahr M, van der Zee D, Luijckx G, et al.
A simulation based approach for improving utilization of thrombolysis in acute brain infarction. *Medical Care* 2013;51(12):1101-5.

25 Wahlgren N, Ahmed N, Davalos A, et al. Thrombolysis with alteplase for acute ischaemic stroke in the Safe Implementation of Thrombolysis in Stroke-Monitoring Study (SITS-MOST): an observational study. *Lancet* 2007;369:275-82 doi:10.1016/S0140-6736(07)60149-4.

26 Law AM. ExpertFit Version 8 User's Guide. Tuscon, Arizona: Averill M. Law & Associates 2011.

For peer review only

27 Salisbury HR, Banks BJ, Footitt DR, et al. Delay in presentation of patients with acute stroke to hospital in Oxford. *QJM* 1998;91:635-40.

28 Carroll C, Hobart J, Fox C, et al. Stroke in Devon: knowledge was good, but action was poor. *J Neurol Neurosurg Psychiatry* 2004;75:567-71.

29 Barsan WG, Brott TG, Broderick JP, et al. Urgent therapy for acute stroke. Effects of a stroke trial on untreated patients. *Stroke* 1994;25:2132-7.

30 Chen NC, Hsieh MJ, Tang SC, et al. Factors associated with use of emergency medical services in patients with acute stroke. *Am J Emerg Med* 2013;31:788-91 doi:10.1016/j.ajem.2013.01.019 [doi].

31 Powers WJ, Rabinstein AA, Ackerson T, et al. 2018 Guidelines for the Early Management of Patients With Acute Ischemic Stroke: A Guideline for Healthcare Professionals From the American Heart Association/American Stroke Association. *Stroke* 2018;49:e46-e110 doi:10.1161/STR.0000000000000158 [doi].

32 Fonarow GC, Smith EE, Saver JL, et al. Timeliness of tissue-type plasminogen activator therapy in acute ischemic stroke: patient characteristics, hospital factors, and outcomes associated with door-to-needle times within 60 minutes. *Circulation* 2011;123:750-8 doi:10.1161/CIRCULATIONAHA.110.974675.

33 Zinkstok SM, Beenen LF, Luitse JS, et al. Thrombolysis in Stroke within 30 Minutes: Results of the Acute Brain Care Intervention Study. *PLoS One* 2016;11:e0166668 doi:10.1371/journal.pone.0166668 [doi].

34 Meretoja A, Weir L, Ugalde M, et al. Helsinki model cut stroke thrombolysis delays to 25 minutes in Melbourne in only 4 months. *Neurology* 2013;81(12):1071-6 doi:10.1212/WNL.0b013e3182a4a4d2.

35 Lees KR, Bluhmki E, von Kummer R, et al. Time to treatment with intravenous alteplase and outcome in stroke: an updated pooled analysis of ECASS, ATLANTIS, NINDS, and EPITHET trials. *Lancet* 2010;375:1695-703 doi:10.1016/S0140-6736(10)60491-6.

36 Atkins DL, Everson-Stewart S, Sears GK, et al. Epidemiology and outcomes from out-of-hospital cardiac arrest in children: the Resuscitation Outcomes Consortium Epistry-Cardiac Arrest. *Circulation* 2009;119:1484-91 doi:10.1161/CIRCULATIONAHA.108.802678.

37 Jauch EC, Saver JL, Adams HP,Jr, et al. Guidelines for the early management of patients with acute ischemic stroke: a guideline for healthcare professionals from the American Heart Association/American Stroke Association. *Stroke* 2013;44:870-947 doi:10.1161/STR.0b013e318284056a [doi].

38 Acker JE,3rd, Pancioli AM, Crocco TJ, et al. Implementation strategies for emergency medical services within stroke systems of care: a policy statement from the American Heart Association/American Stroke Association Expert Panel on Emergency Medical Services Systems and the Stroke Council. *Stroke* 2007;38:3097-115 doi:10.1161/STROKEAHA.107.186094.

For peer review only

39 Boode B, Welzen V, Franke C, et al. Estimating the number of stroke patients eligible for thrombolytic treatment if delay could be avoided. *Cerebrovasc Dis* 2007;23:294-8 doi:10.1159/000098330.

40 Lahr MM, van der Zee DJ, Luijckx GJ, et al. Improving acute stroke services in the Netherlands. *BMJ* 2014;348:g3957 doi:10.1136/bmj.g3957 [doi].

41 Monks T, Pitt M, Stein K, et al. Hyperacute stroke care and NHS England's business plan. *BMJ* 2014;348:g3049 doi:10.1136/bmj.g3049 [doi].

42 Caminiti C, Schulz P, Marcomini B, et al. Development of an education campaign to reduce delays in pre-hospital response to stroke. *BMC Emerg Med* 2017;17:20,017-0130-9 doi:10.1186/s12873-017-0130-9 [doi].

Figure legends:

Figure 1. Acute stroke pathway: description of activities.

Figure 2. Summary of best practices and relative gap (%) between baseline model and optimised settings. This figure depicts the total relative gap in IVT rates between the baseline model and the optimised scenarios (i.e. time delays set at zero and diagnostics at 100%). The colored bars represent the relative gains in IVT rates, per best practice (A-D) in each of the improvement domains. Also the contribution of combined best practices are considered.

For peer review only

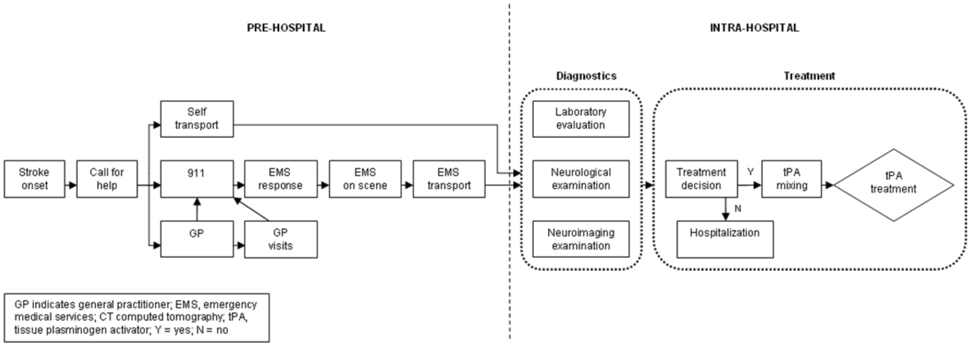

Figure 1. Acute stroke pathway: description of activities.

324x166mm (96 x 96 DPI)

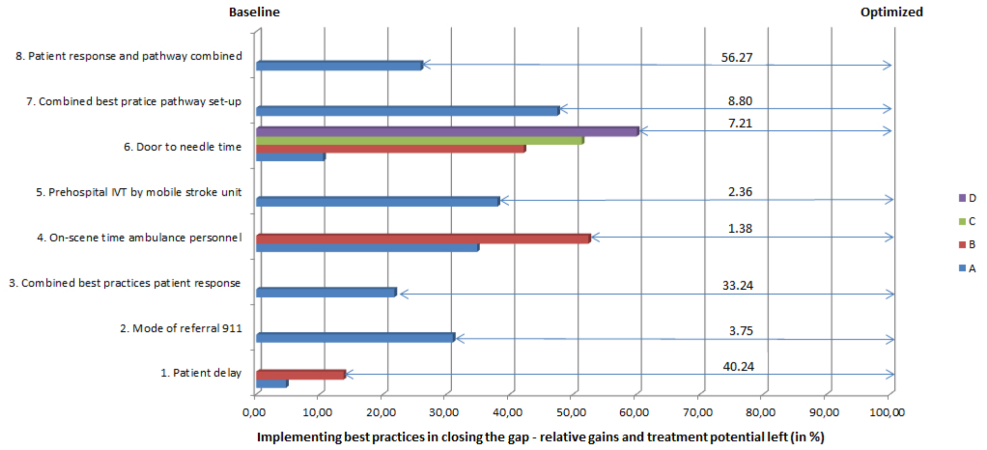

Figure 2. Summary of best practices and relative gap (%) between baseline model and optimized settings. This figure depicts the total relative gap in IVT rates between the baseline model and the optimized scenarios (i.e. time delays set at zero and diagnostics at 100%). The colored bars represent the relative gains in IVT rates, per best practice (A-D) in each of the improvement domains. Also the contribution of combined best practices are considered.

274x166mm (96 x 96 DPI)

BMJ Open

Optimizing acute stroke care organization: a simulation study to assess the potential to increase intravenous thrombolysis rates and patient gains

Journal:	*BMJ Open*
Manuscript ID	bmjopen-2019-032780.R1
Article Type:	Original research
Date Submitted by the Author:	21-Oct-2019
Complete List of Authors:	Lahr, Maarten ; University of Groningen, University Medical Center Groningen, Health Technology Assessment, Department of Epidemiology<br>van der Zee, Durk-Jouke; Rijksuniversiteit Groningen Faculteit Economie en Bedrijfskunde, Operations<br>Luijckx, Gert-Jan; University of Groningen, University Medical Center Groningen, Neurology<br>Buskens, Erik; University of Groningen, University Medical Center Groningen, Epidemiology; University of Groningen, Faculty of Economics and Business, Deparment of Operations
**Primary Subject Heading**:	Neurology
Secondary Subject Heading:	Epidemiology, Health services research
Keywords:	Stroke < NEUROLOGY, Organisation of health services < HEALTH SERVICES ADMINISTRATION & MANAGEMENT, EPIDEMIOLOGY

SCHOLARONE™
Manuscripts

Optimizing acute stroke care organization: a simulation study to assess the potential to increase intravenous thrombolysis rates and patient gains

Maarten M.H. Lahr[1] Ph.D., Durk-Jouke van der Zee[2] Ph.D., Gert-Jan Luijckx[3] M.D., Ph.D., Erik Buskens[1,2] M.D., Ph.D.

[1] *Health Technology Assessment, Department of Epidemiology, University of Groningen, University Medical Center Groningen, Groningen, The Netherlands*

[2] *Department of Operations, Faculty of Economics & Business, University of Groningen, Groningen, The Netherlands*

[3] *Department of Neurology, University of Groningen, University Medical Center Groningen, Groningen, The Netherlands*

Corresponding Author:

Maarten M.H. Lahr

University Medical Center Groningen, Department of Epidemiology

P.O. Box 30001. 9700 RB Groningen, The Netherlands

Telephone: + 31 50 361 43 86

E-mail: m.m.h.lahr@umcg.nl

Word count paper: 3490 (max 3500 excl. abstract, references, tables, and figure legends)

Word count abstract: 243 (max 250)

Number of text pages: 13

Number of references: 44 (max 50)

For peer review only

Number of figures/tables: 4 (max 5 incl. tables)

Brief title: Optimizing acute stroke care systems

Complete author information:

Durk-Jouke van der Zee, PhD. Correspondence address: University of Groningen, Faculty of Economics & Business, Department of Operations. 9747 AE Groningen, The Netherlands. Email: d.j.van.der.zee@rug.nl

Gert-Jan Luijckx, MD, PhD. Correspondence address: University Medical Center Groningen, Department of Neurology. 9713 GZ Groningen, The Netherlands. Email: g.j.luijckx@umcg.nl

Erik Buskens, MP, PhD. Correspondence address: University Medical Center Groningen, Department of Epidemiology. 9713 GZ Groningen, The Netherlands. Email: e.buskens@umcg.nl

Key words: acute stroke, thrombolysis, organizational model, simulation modeling, quality improvement.

Conflict of interest: the authors declare no conflict of interest.

Acknowledgements: None.

Abstract

For peer review only

Objectives To assess potential increases in Intravenous Thrombolysis (IVT) rates given particular interventions in the stroke care pathway.

Design Simulation modeling was used to compare the performance of the current pathway, best practices based on literature review, and an optimized model.

Setting Four hospitals located in the North of the Netherlands, as part of a centralized organizational model.

Participants Ischemic stroke patients prospectively ascertained from February to August 2010.

Intervention The interventions investigated included efforts aimed at patient response and mode of referral, prehospital triage, and intra-hospital delays.

Primary and secondary outcome measures The primary outcome measure was thrombolysis utilization. Secondary measures were onset-treatment time (OTT) and the proportion of patients with excellent functional outcome (mRS 0-1) at 90 days.**Results**

Of 280 patients with ischemic stroke, 125 (44.6%) arrived at the hospital within 4.5 hours, and 61 (21.8%) received IVT. The largest improvements in IVT treatment rates, OTT, and the proportion of patients with mRS scores of 0-1 can be expected when patient response is limited to 15 minutes (IVT rate +5.8%; OTT -6 minutes; excellent mRS scores +0.2%), door-to-needle time to 20 minutes (IVT rate +4.8%; OTT -28 minutes; excellent mRS scores +3.2%), and 911 calls are increased to 60% (IVT rate +2.9%; OTT -2 minutes; excellent mRS scores +0.2%). The combined implementation of all potential best practices could increase IVT rates by 19.7% and reduce OTT by 56 minutes.

Conclusions

Improving IVT rates to well above 30% appears possible if all known best practices are implemented.

Strengths and limitations of the study

For peer review only

- The simulation modelling study included a comprehensive collection of patient level data from both the pre- and intrahospital acute stroke pathway.
- The generic modelling approach adopted could be extended to include patient data from other stroke care systems.
- Costs items associated with the proposed interventions could not be collected and controlled for.
- Estimations of time intervals used for model building might have changed over time.

For peer review only

Introduction

Intravenous thrombolysis (IVT) is an effective therapy for acute ischemic stroke up to 4.5 hours after the onset of symptoms.[1, 2] This therapy is substantially underused, however, with 8-10% of all stroke patients worldwide currently receiving IVT.[3, 4] In contrast, treatment rates of up to 35% have been achieved in optimized settings.[5, 6] The organization of stroke care is an important factor in realizing timely hospital arrival and treatment.[7, 8] The centralization of care at designated stroke centers has been demonstrated to increase the proportion of patients arriving at the hospital in time for acute treatment.[9-11] Given the substantial decrease in the benefit of treatment with increasing time delays, further efforts to expedite hospital arrival and subsequent treatment remain of crucial importance.[12, 13]

Various studies have investigated factors associated with efficiency in each part of the acute stroke pathway. Although it has been generally established that delay on the part of patients and/or bystanders is a primary factor in delaying hospital arrival,[14] interventions aimed at improving optimal response by calling 911 immediately have exhibited varying success, and many lack sustained implementation[15] (as is the case in other domains of medicine as well).[16] Ambulance transportation to hospitals that offer acute treatment is associated with shorter onset-to-door times, reductions in intra-hospital delays, and increases in treatment rates.[15, 17, 18] The provision of acute treatment by a Mobile Stroke Unit (MSU) before hospital arrival has been identified as a promising method for reducing time to IVT. [19]Another widely studied topic concerns reducing the time between hospital arrival and treatment (door-to-needle time, or DTN), with reported DTN times as low as 20 minutes.[20]

The aforementioned studies have demonstrated clear benefits in terms of time saved. They nevertheless lack a broader overview of pathway set-up and its performance. Instead of addressing the solution space as a whole, they target isolated elements of the stroke pathway. = The lack of a broader overview is due in part to the predominant use of randomized controlled trials (RCT) as the main research vehicle. Given the effort involved in their set-up, RCT studies

For peer review only

focus predominantly on separate and singular elements of pathway performance. They may therefore be less suitable for investigating complex care systems (e.g., acute stroke treatment). In particular, timely hospital arrival and the treatment of acute stroke patients relies on a series of intertwined activities concerning patient diagnostics and transportation.

One potential alternative methodology is simulation modeling. Proceeding from a detailed description of both pre-hospital and intra-hospital time delays and diagnostics, an accurate representation of pathway performance can be developed *in silico*, including the validation of IVT rates, time to treatment, patient outcomes (as measured by the modified Rankin Scale or mRS), and other clinically relevant outcome measures.[21, 22] This approach would allow the examination of all time delays and diagnostic steps, thereby providing clinicians and policymakers with an accurate overview of obstacles currently existing within care pathways. In addition, scenarios for hypothetical approaches to improvement based on clinical guidelines, literature observations, and/or expert opinion can be modeled and studied for their cumulative effects on relevant clinical outcome measures.[23, 24]

The aims of this simulation-modeling study were (1) to estimate the cumulative potential for improving IVT utilization by implementing best practices on the organization of the stroke pathway and, subsequently, (2) to explore areas in which further improvement is needed in order to achieve a fully optimized setting, in addition to identifying obstacles to such optimization.

Methods

This article is based on a six-month, prospective, multi-center study performed in a centralized organizational stroke-care setting in the north of the Netherlands from February through July 2010.[9] Patient-level data were collected on time delays and diagnostics, thereby providing detailed insight into patient flow and potential obstacles in both the pre-hospital and intra-

For peer review only

hospital pathways (Table 1). A schematic overview of patient flow and the steps included in the analyses is presented in Figure 1.

Table 1. Descriptive statistics of activity durations and diagnostics

Number of patients	280
Age in years (SD)	70 (14)
Male (%)	156 (56)
**Patient responsiveness**	
Time from symptom onset to call for help, valid cases (%)	152 (54)
Median, minutes (IQR)	41 (5-130)
Mode of referral (%)	
General practitioner	129 (46)
911	84 (30)
Self-referral	60 (21)
In-hospital patients	7 (3)
**Pathway set-up**	
Transported by EMS (%)	213 (76)
Response time, median (min)	9 (7-12)
On scene time, median (min)	20 (15-25)
Transportation time, median (min)	17 (9-22)
Median time from hospital arrival to neurological examination, min (IQR)	2 (0-0)
Median time from hospital arrival to CT examination min (IQR)	12 (6-15)
Median time from hospital arrival to laboratory examination, min (IQR)	32 (27-37)
Median door to IVT time, min (IQR)	35 (25-45)

SD indicates standard deviation; IQR, Interquartile Range; EMS, Emergency Medical Services; CT, Computed Tomography; IVT, Intravenous Thrombolysis.

Setting and participants

The centralized organizational setting consisted of four hospitals in the northern region of the Netherlands, with IVT being administered only in the University Medical Center Groningen (UMCG). Together with the other three community hospitals, general practitioner (GP) offices, and emergency medical services (EMS), arrangements were made to transport presumed stroke

For peer review only

victims immediately to the UMCG, thus bypassing community hospitals that might have been located closer to the patient's location. The international protocol for IVT (adjusted ECASS III[25]) and the regional protocol for pre-hospital management were followed. The region addressed in this study comprises approximately 580,000 inhabitants, with a population density of 250 inhabitants per square kilometer. The study population consisted of ischemic stroke patients admitted to all four hospitals between February and August 2010. Case ascertainment was confirmed by the final hospital discharge diagnosis of ischemic stroke, thereby excluding stroke mimics.

The data collected included information on time delays and diagnostics along the entire stroke chain. Time delays included the time from symptom onset to call for help, delay at first response (GP or EMS services), EMS transport times, and intra-hospital diagnostics, which included time from hospital arrival to neurological examination, Computed Tomography (CT) scan, laboratory testing, and IVT.

Simulation model

The current study was performed using a previously validated simulation model.[24] More detailed information on simulation modeling methodology, input parameters, model and model data used may be found in a supplementary file. This model was populated with patient-level data from a previous observational study.[9] The model was validated by comparing IVT treatment percentages and OTT to those reported in the observational study. The next step consisted of developing scenarios in which alternative pathway set-ups, associated time delays, and diagnostics were imputed based on literature observations, clinical guidelines, and expert opinion. Using the simulation model, hypothetical patients were passed through the system, estimating the impact of intervening at various points in the acute stroke pathway. Interventions

For peer review only

were modeled by changing the underlying statistical distributions to redistribute patients across time delays and diagnostics.[26]

Scenarios

We used simulation modeling to investigate the effects of changing pathway set-ups, based on three models: (1) a baseline model of acute stroke care; (2) a model reflecting best practices, based on a review of the current literature, clinical guidelines, and expert opinion; and (3) an optimized model. Interventions were selected according to the obstacles identified in the current centralized organizational model. Obstacles within the study setting included delayed emergency response by patients following symptom onset, mode of referral (GP or 911), time spent on the scene by ambulance personnel, and intra-hospital delays. The simulation model was used to perform hypothetical interventions in the pathway to calculate clinically relevant outcomes.[24] The outcomes were compared to the baseline performance of the current system to estimate the potential for improvement, to optimize system performance, and to identify obstacles that have yet to be overcome.

<u>Baseline</u>

The baseline model describes the performance of the centralized organizational model for acute stroke care, as described in the previous observational study.[9] A description of time delays and diagnostics along each step of the pathway is provided in Table 1.

<u>Best practice</u>

The scenarios that were investigated are described in Table 2.

Patient responsiveness: We estimated the relative impact of reducing the time between stroke onset and call for help by patients, their families, and/or bystanders by adjusting the distribution

For peer review only

of their response times by a factor equal to the quotient of the respective median response times reported for best practices (15-30 minutes)[27, 28] and the baseline scenario (41 minutes).

Mode of referral: We modeled a scenario in which patients, their families, and/or bystanders predominantly (60%) chose 911 as the mode of entry.[29, 30]

Time spent on the scene by ambulance personnel: In this scenario, we modeled the time spent on the scene by ambulance personnel following a 911 call by imposing an upper boundary on on-scene delay. Based on the guidelines of the American Stroke Association, the time spent on the scene should be no more than 15 minutes.[31] We also modeled the implementation of a "scoop and run protocol," which includes prompt transport (≤ 10 minutes) with initial management efforts, while postponing elaborate triage.

Pre-hospital treatment by MSU: In this scenario, we modeled pre-hospital IVT provided by a MSU by adjusting only the transport time of the ambulance. Assuming that the MSU would be stationed at the hospital, we considered only the transportation time from the hospital to the patient scene.

For peer review only

Table 2. Overview of scenarios.

Patient responsiveness	Baseline	Best practice (scenario)	Study	Optimized (scenario)
Patient delay, median (min)	41	30 (1A)<br>15 (1B)	Salisbury et al. (1998)<br>Carrol et al. (2004)	0 (1C)
Mode of referral 911 (%)	30	60 (2A)	Barsan et al. (1994)<br>Chen et al. (2013)	100 (2B)
**Pathway set-up**				
Transportation EMS				
Response time, median (min)	9	-	-	0 (5)
On scene time, median (min)	20	15 (6A)<br>10 (6B)	Jauch et al. (2013), Acker et al. (2007)<br>Atkins et al. (2009)	0 (6C)
Transportation time, median (min)	17	-		0
Prehospital IVT by MSU	-	Transportation time = 0 (7A)	-	Response time = 0 (7B)<br>Transportation time = 0
Door-to-IVT time, median (min)	35	30 (8A)<br>25 (8B)<br>20 (8C)	Zinkstok et al. (2016)<br>Meretoja et al. (2013)<br>Meretoja et al. (2012)	0 (8D)

EMS indicates emergency medical services; IVT, intravenous thrombolysis; MSU, Mobile Stroke Unit.
Scenarios reflecting combined interventions not displayed here include: scenario 3 combined best practices patient responsiveness (scenarios 1B+2A) and scenario 4 reflects optimised patient responsiveness (scenarios 1C+2B); scenario 9 combined best practices pathway set-up (scenarios 6B+7A+8C) and scenario 10 reflects optimised pathway set-up (scenarios 5+6C+7B+8D); Scenario 11 combined best practices patient responsiveness and pathway set-up (scenarios 3+9) and scenario 12 reflects optimised patient responsiveness and pathway set-up (scenarios 4+10).

For peer review only

Intra-hospital processes: Intra-hospital delays comprise all activities performed between arrival at the hospital until the start of IVT (door-to-needle time; see Figure 1). Based on the guidelines of the American Stroke Association, door-to-needle time should be no more than 60 minutes.[32] Evidence from clinical practice suggests that DTN times within a range of 20 to 30 minutes are attainable.[20, 33, 34]

Combined best-practice scenarios

Three scenarios were performed in which best practices were combined for patient responsiveness and pathway set-up. Patient responsiveness was modeled by combining patient response with the mode of referral following stroke onset. Pathway set-up was modeled by combining pre-hospital and intra-hospital best practices. A third scenario combines best practices for patient responsiveness and pathway set-up.

Optimized scenarios

Additional scenarios were defined to interpret findings on the effects of the implementation of best practices (or combinations thereof) by generating upper boundaries to pathway performance, thereby building on unrealistically optimistic assumptions (see the last column in Table 2). For the optimized scenarios, we extrapolated best practices by setting the parameter for associated time delays to 0 or by setting the diagnostic quality parameter to 100%.

Outcome measures

The primary outcome measure was the proportion of patients treated with IVT. Secondary outcome measures were the total process time (onset-to-treatment time), IVT within various time intervals (0-90, 91-180, and 181-270 minutes), the proportion of patients with favorable

For peer review only

outcomes at 90 days (modified Rankin Scale 0-1), and additional healthy life days (calculated using OTT estimates).[12, 35]

Analysis

For each of the scenarios described above, we calculated new hypothetical IVT treatment rates and secondary outcome measures, based on the number of patients arriving in time for acute treatment at the hospital (i.e., within 4 hours after symptom onset), the number treated with IVT, and the time to treatment. Chi-square tests were used to compare categorical variables.

Informed consent

Informed consent was obtained from all subjects participating in the prospective study and extended for the current simulation study. As such no additional approval from our local ethics committee was required.

Data availability statement

The authors state that anonymized data will be shared by request from any qualified investigator.

Patient and public involvement

Patients and public were not involved in the design of this study. For this modelling study non-identifiable patient data was used. Study results will be disseminated through poster presentations and publication in peer-reviewed journals.

Results

For peer review only

This study reflects the experiences of 280 ischemic stroke patients referred to the UMCG and three community hospitals, as part of a centralized organizational model. Baseline and demographic characteristics are described in Table 1. In all, 125 (44.6%) patients arrived at the hospital within 4.5 hours, 61 (21.8%) received IVT, and the median OTT was 127 minutes. Of the patients receiving IVT, 17.0% were treated within 90 minutes of symptom onset. Patient delay, intra-hospital delays, and mode of referral (GP or 911) were identified as the greatest obstacles to receiving IVT (Table 1).

Simulation experiments

The results of the simulation experiments are presented in Table 3.

Patient responsiveness: If patients had contacted emergency services sooner (i.e., within 30 and 15 minutes), up to 27.6% (CI 26.7% – 28.4%) of the total population would have been treated with IVT (Table 3, Scenarios 1A and 1B), and the OTT would have been reduced to 122 minutes (CI 121 – 124).[27, 28]

Assuming a patient delay of 0 minutes (Table 3, Scenario 1C), 64.0% of the total population would have been treated with IVT, and the OTT would have been reduced to 92 minutes (CI 91 – 92).

Mode of referral: Assuming 60% of all patients contacting 911 immediately following stroke onset (Table 3, Scenario 2A) increased the IVT rate to 24.6% (CI 23.8% – 25.5%) and reduced the OTT to 127 minutes (CI 125 – 128).[29]

If all patients (100%) had called 911 (Table 3, Scenario 2B), the IVT rates would have increased further to 28.4% (CI 27.5% – 29.3%), and the OTT would have been reduced to 124 minutes (CI 123 – 126).

For peer review only

Time spent on the scene by ambulance personnel: Shortening the time spent on the scene to 15 and 10 minutes (Table 3, Scenarios 6A and 6B),[36] increased the IVT treatment rate up to 23.3% (CI 22.4% – 24.1%) and reduced the OTT to 121 minutes (CI 120 – 123).[37, 38]

For peer review only

Table 3. Re-configuration centralised model: results simulation experiments.

	IVT rate (95% CI)	OTT minutes (95% CI)	IVT 0-1.5 hours	IVT 1.5-3.0 hours	IVT 3.0-4.5 hours	mRS 0-1†	Extra healthy days‡
**Scenario**							
0.Current practice	21.8% (20.9% - 22.6%)	129 (127 – 130)	17.0%	70.9%	12.1%	12.5%	
**Patient responsiveness**							
1.Patient delay							
A. Reduced to 30 minutes	23.7% (22.9% - 24.6%)	127 (125 – 129)	16.9%	72.2%	10.9%	12.6%	2.9
B. Reduced to 15 minutes	27.6% (26.7% - 28.4%)*	122 (121 – 124)	16.5%	76.7%	6.8%	12.7%	11.3
C. Reduced to 0 minutes	64.0% (63.0% - 64.9%)*	92 (91 – 92)	45.7%	54.1%	0.2%	16.2%	66.9
2. Mode of referral 911							
A. Increased to 60%	24.6% (23.8% - 25.5%)	127 (125 – 128)	18.8%	70.0%	11.2%	12.8%	3.9
B. Increased to 100%	28.4% (27.5% - 29.3%)*	124 (123 – 126)	18.3%	71.4%	10.3%	12.7%	7.8
3. Combined best practices patient responsiveness (1B+2A)	31.0% (30.1% - 31.9%)*	120 (118 – 121)	19.2%	74.4%	6.4%	13.0%	16.3
4. Optimised patient responsiveness (1C + 2B)	64.3% (63.3% - 65.2%)*	98 (97 – 98)	36.2%	63.7%	0.1%	15.1%	55.7
**Pathway set-up**							
5. Response time first responders reduced to 0 minutes	23.3% (22.4% - 24.1%)	121 (119 - 122)	21.8%	68.9%	9.3%	13.2%	14.4
6. On scene time ambulance personnel							
A. Reduced to 15 minutes	22.8% (21.9% - 23.6%)	124 (122 – 126)	19.4%	69.9%	10.7%	12.8%	8.6
B. Reduced to 10 minutes	23.3% (22.4% - 24.1%)	121 (120 – 123)	21.7%	68.5%	9.8%	13.1%	13.4
C. Reduced to 0 minutes	24.7% (23.8% - 25.5%)	114 (112 – 116)	31.4%	60.4%	8.2%	14.3%	26.3
7. Prehospital IVT by MSU							
A. Transportation time reduced to 0 minutes	23.2% (22.4% - 24.0%)	121 (120 – 123)	22.8%	67.4%	9.8%	13.2%	13.1
B. All ambulance delays reduced to 0 minutes	25.6% (24.7% – 26.4%)	109 (107 – 110)	38.6%	54.3%	7.0%	15.1%	35.8
8. Door to IVT times							
A. Reduced to 30 minutes	25.2% (24.3% - 26.0%)	110 (108 – 111)	34.3%	57.7%	8.0%	14.6%	34.4
B. Reduced to 25 minutes	25.9% (25.0% - 26.8%)	106 (104 – 107)	39.3%	53.2%	7.5%	15.2%	41.5
C. Reduced to 20 minutes	26.6% (25.7% - 27.4%)	101 (99 – 103)	44.2%	48.8%	7.0%	15.7%	49.8
D. Reduced to 0 minutes	29.8% (28.9% - 30.7%)*	83 (81 – 85)	62.9%	32.1%	5.0%	17.9%	81.9
9. Combined best practices pathway set-up (6B+7A+8C)	30.8% (29.9% - 31.7%)*	80 (79 – 82)	68.9%	26.3%	4.8%	18.6%	87.3
10. Optimised pathway set-up (5 + 6C + 7B + 8D)	38.5% (37.6% - 39.5%)*	39 (37 – 40)	86.0%	11.3%	2.7%	20.6%	161.7
**Patient responsiveness and pathway set-up**							

For peer review only

For peer review only - http://bmjopen.bmj.com/site/about/guidelines.xhtml

11. Combined best practices patient responsiveness and pathway set-up (3+9)	41.5% (40.5% - 42.4%)*	73 (72 – 74)	77.4%	20.5%	2.2%	19.6%	99.9
12. Optimised patient responsiveness and pathway set-up (4+10)	97.7% (97.4% – 98.0%)*	0 (0 – 0)	100.0%	0.0%	0.0%	22.2%	231.6

IVT indicates intravenous thrombolysis; CI, confidence interval; OTT, onset-treatment-time; tPA indicates tissue plasminogen activator; mRS, modified rankin scale; MSU, mobile stroke unit.

* Sig. <0.05

† Indicates the proportion of patients with good outcome (mRS 0-1) ascribed to treatment with thrombolysis.[33]

‡ Indicated the number of additional days in healthy life by minute reduction in OTT.[1]

For peer review only

Reducing time on the scene to 0 minutes resulted in a projected IVT rate of 24.7% (CI 23.8% – 25.5%) (Table 3, Scenario 6C) and a decrease in the OTT to 114 minutes (CI 112 – 116).

Pre-hospital treatment by Mobile Stroke Unit (MSU): In this set-up, 23.2% (CI 22.4% – 24.0%) of patients would have been treated with IVT (Table 3, Scenario 7A) and OTT would have been reduced to 121 minutes (CI 120 – 123).

The elimination of both the response time and transportation time of the MSU (Table 3, Scenario 7B) resulted in a projected 25.6% (CI 24.7% - 26.4%) of patients that could have received IVT and a reduction of the OTT to 109 minutes (CI 107 – 110).

Intra-hospital processes: If the DTN time had been shortened to a maximum of 30 and 20 minutes (Table 3, Scenarios 8A-C), up to 26.6% (CI 25.7% - 27.4%) of the total population would have been treated with IVT, and the OTT would have been reduced to 101 minutes (CI 99 – 103).

If the DTN had been reduced to 0 minutes (Table 3, Scenario 8D), 29.8% (CI 28.9 – 30.7%) of all patients would have been treated with IVT, and the OTT would have been reduced to 83 minutes (CI 81 – 85).

Combined best practice scenarios

Combining best practices for patient responsiveness and pathway set-up (Table 3, scenario's 3,9,11) resulted in up to 41.5% (CI 40.5% - 42.4%) of all patients being treated with IVT and reduced the OTT to 73 minutes (CI 72 – 74).

Optimized scenarios

Assuming optimized patient responsiveness (i.e. all patients calling 911 immediately following stroke onset; Table 3, Scenario 4) resulted in 64.3% of the total population (CI 63.3% – 65.2%)

For peer review only

being treated with IVT and reduced the OTT to 98 minutes (CI 97 – 98). The optimization of pathway set-up (Table 3, Scenario 10) resulted in 38.5% (CI 37.6% – 39.5%) of all patients receiving IVT and reduced the OTT to 39 minutes (CI 37 – 40). The combination of all optimized scenarios (Table 3, Scenario 12) resulted in a cumulative total of 97.7% (CI 97.4% - 98.0%) of all patients being treated with IVT and reduced the OTT to 0 minutes.

Discussion

This study demonstrates that IVT treatment rates above 30% would be possible if best practices were to be implemented within our setting. We modeled several scenarios to generate insight into the potential for quality improvements in our acute stroke chain of care. Although improvements in patient responsiveness would yield the largest potential gains within our pathway, even modest changes in this regard are likely to be challenging and costly to achieve.[16] In contrast, improvements in other areas (e.g., intra-hospital delays and time spent by ambulance personnel at the patient's location) might be easier to achieve and would still lead to clinically relevant increases in IVT rates. As indicated in previous studies, even small reductions in time to treatment with IVT are associated with considerable increases in the length of healthy life, and they may require only relatively simple organizational changes involving minimal effort at little or no cost.[12]

The results of our study may be useful as a guide for prioritizing interventions along the acute stroke pathway and for estimating their potential impact on the effectiveness of the pathway. A simulation-based approach, as presented in this paper, can be instrumental in facilitating a broad overview of the set-up and performance of stroke pathways. This could provide clinicians and policymakers with speedy answers – at little effort or cost – concerning how new or widely advocated practices could be used to improve their pathways, thus allowing them to direct investments to the interventions that matter most. It thus has the potential to

For peer review only

replace RCT studies or serve as a precursor to a focused RCT, which could be scoped as the net result of a simulation approach.

In our study, we observed the greatest effects on IVT rates, time to treatment, and patient outcomes after improving the responsiveness of patients and/or bystanders by reducing the time from symptom onset to the call for help, thereby expediting intra-hospital care services and by increasing the number of 911 calls made by patients or bystanders. In contrast, a scenario in which pre-hospital transportation delays were reduced by the implementation of a MSU resulted in only moderate effects. Combining all of the best-practice scenarios resulted in a maximum of 41.5% of patients being treated with IVT. This is substantially higher than current benchmark figures on clinical practice, which suggest a maximum IVT rate of around 35%.[6, 39]

Proceeding from a scenario in which best practices have been implemented, remaining challenges include realizing further decreases in time delays in both the pre-hospital and intra-hospital phases. The feasibility of such initiatives in clinical practice might be limited in the short term, however, given the current lack of evidence concerning solutions for further expediting care and logistics services at reasonable costs. For example, the goal of reducing time spent on the scene by ambulance personnel to less than 10 minutes or reducing door-to-needle time to less than 20 minutes would probably be unrealistic, given the need to handle and observe the patient, to complete diagnostic tests, and to interpret findings. Although further improvements in the proportion of patients calling 911 directly following symptom onset could potentially result in further increases in IVT rates, they would also necessitate large-scale and repetitive publicity campaigns comparable to those launched to raise public awareness on stroke symptoms and how to act.

The organizational model for acute stroke care delivery is currently receiving a great deal of attention in the Netherlands, as well as beyond.[40, 41] The emergence of endovascular treatment (EVT) for patients facing large-vessel occlusions has opened up a whole new

For peer review only

dimension in terms of acute stroke pathway set-up and patient logistics. Following IVT, eligible patients must now undergo additional diagnostic evaluation (e.g., CT angiography and perfusion CT), followed by such EVT treatment modalities as groin punctures and initial attempt at clot retrieval with the device up to the angioseal following successful recanalization. In addition, within the current “drip-and-ship” treatment paradigm, eligible patients may initially be admitted to community hospitals before being transferred to comprehensive stroke centers with EVT capacity, thereby further increasing the number of logistical steps. Given the time-sensitive nature of acute stroke interventions, this extension of the pathway necessitates the re-organization of acute stroke care within regions and settings. In this respect, simulation modeling could facilitate insight into the complex interplay of separate elements of the pathway. Currently positioned as a follow-up treatment by current guidelines, availability of EVT does not change the need for optimizing utilization of IVT, nor does it impact the acute stroke pathway set-up for IVT. Moreover, the subgroup of patients eligible for EVT is relatively small, around 7% of all stroke patients.[42]

Our study is subject to several limitations. First, our simulation models did not consider the response of GPs when contacted as first responders. Although this has been signaled as an issue for delays in hospital arrival for patients,[43] no studies on best practices were identified in the literature. Second, because the costs and cost-effectiveness associated with pathway improvements in our setting were not estimated in our model, it was not possible to control for them. Third, the results of our findings might not be generalizable to other settings, due to the unique position of the UMCG, which serves as a stroke center in a centralized organizational model deployed within its region. As noted in a previous publication, however, the generic modeling approach adopted in this study could be extended to include a description of a decentralized organizational model, which receives IVT candidates within its own catchment areas.[23] Also, because patients were enrolled in the observational study back in 2010 these

For peer review only

results will not fully reflect current practice. However, review of internal databases show that IVT treatment percentages have remained largely stable over the last years, fluctuating around 25% with a DTN time of around 35 minutes. EMS response times have remained constant over the years.[44] Future activities should be aimed at extending the simulation-based approach to include the drip-and-ship model currently employed in acute stroke treatment (e.g., EVT).

Conclusions

The results of this study indicate that the cumulative effects of implementing best practices on the organization of stroke care would clearly exceed current benchmarks for treatment rates. Remaining obstacles might be difficult to overcome given the limited availability of solution to further expedite care and logistical services at tolerable costs. A broader overview facilitated by simulation is suggested as instrumental in supporting decision-makers and clinicians in their efforts to evaluate the set-up and performance of acute stroke pathways.

Contributor statement ML and DJZ designed the study, performed the experiments and analyzed the data. ML drafted the manuscript, DJZ, GJL and EB critically revised the manuscript for intellectual content and approved the final version of the manuscript for publication.

Competing interests None declared

Funding Not applicable

Data sharing statement

For peer review only

The data used and analysed in the current study is available from the corresponding author upon request.

References

1 . Tissue plasminogen activator for acute ischemic stroke. The National Institute of Neurological Disorders and Stroke rt-PA Stroke Study Group. *N Engl J Med* 1995;333:1581-7 doi:10.1056/NEJM199512143332401.

2 Hacke W, Kaste M, Bluhmki E, et al. Thrombolysis with alteplase 3 to 4.5 hours after acute ischemic stroke. *N Engl J Med* 2008;359:1317-29 doi:10.1056/NEJMoa0804656.

3 Bauer A, Limburg M, Visser MC. Variation in clinical practice of intravenous thrombolysis in stroke in the Netherlands. *Cerebrovasc Dis Extra* 2013;3:74-7 doi:10.1159/000350707; 10.1159/000350707.

4 Adeoye O, Hornung R, Khatri P, et al. Recombinant tissue-type plasminogen activator use for ischemic stroke in the United States: a doubling of treatment rates over the course of 5 years. *Stroke* 2011;42:1952-5 doi:10.1161/STROKEAHA.110.612358.

5 Waite K, Silver F, Jaigobin C, et al. Telestroke: a multi-site, emergency-based telemedicine service in Ontario. *J Telemed Telecare* 2006;12:141-5 doi:10.1258/135763306776738611.

6 Stolz E, Hamann GF, Kaps M, et al. Regional differences in acute stroke admission and thrombolysis rates in the German federal state of Hesse. *Dtsch Arztebl Int* 2011;108:607-11 doi:10.3238/arztebl.2011.0607 [doi].

7 Bekelis K, Marth NJ, Wong K, et al. Primary Stroke Center Hospitalization for Elderly Patients With Stroke: Implications for Case Fatality and Travel Times. *JAMA Intern Med* 2016;176:1361-8 doi:10.1001/jamainternmed.2016.3919 [doi].

8 Willeit J, Geley T, Schoch J, et al. Thrombolysis and clinical outcome in patients with stroke after implementation of the Tyrol Stroke Pathway: a retrospective observational study. *Lancet Neurol* 2015;14:48-56 doi:10.1016/S1474-4422(14)70286-8 [doi].

9 Lahr MM, Luijckx GJ, Vroomen PC, et al. Proportion of patients treated with thrombolysis in a centralized versus a decentralized acute stroke care setting. *Stroke* 2012;43:1336-40 doi:10.1161/STROKEAHA.111.641795.

10 Ramsay AI, Morris S, Hoffman A, et al. Effects of Centralizing Acute Stroke Services on Stroke Care Provision in Two Large Metropolitan Areas in England. *Stroke* 2015;46:2244-51 doi:10.1161/STROKEAHA.115.009723 [doi].

11 Morris S, Hunter RM, Ramsay AI, et al. Impact of centralising acute stroke services in English metropolitan areas on mortality and length of hospital stay: difference-in-differences analysis. *BMJ* 2014;349:g4757 doi:10.1136/bmj.g4757 [doi].

For peer review only

12 Meretoja A, Keshtkaran M, Saver JL, et al. Stroke thrombolysis: save a minute, save a day. *Stroke* 2014;45:1053-8 doi:10.1161/STROKEAHA.113.002910 [doi].

13 Tsivgoulis G, Katsanos AH, Kadlecova P, et al. Intravenous thrombolysis for ischemic stroke in the golden hour: propensity-matched analysis from the SITS-EAST registry. *J Neurol* 2017;264:912-20 doi:10.1007/s00415-017-8461-8 [doi].

14 Kleindorfer D, Khoury J, Broderick JP, et al. Temporal trends in public awareness of stroke: warning signs, risk factors, and treatment. *Stroke* 2009;40:2502-6 doi:10.1161/STROKEAHA.109.551861 [doi].

15 Fassbender K, Balucani C, Walter S, et al. Streamlining of prehospital stroke management: the golden hour. *Lancet Neurol* 2013;12:585-96 doi:10.1016/S1474-4422(13)70100-5; 10.1016/S1474-4422(13)70100-5.

16 Wakefield MA, Loken B, Hornik RC. Use of mass media campaigns to change health behaviour. *Lancet* 2010;376:1261-71 doi:10.1016/S0140-6736(10)60809-4.

17 Evenson KR, Foraker RE, Morris DL, et al. A comprehensive review of prehospital and in-hospital delay times in acute stroke care. *Int J Stroke* 2009;4:187-99 doi:10.1111/j.1747-4949.2009.00276.x.

18 Rose KM, Rosamond WD, Huston SL, et al. Predictors of time from hospital arrival to initial brain-imaging among suspected stroke patients: the North Carolina Collaborative Stroke Registry. *Stroke* 2008;39:3262-7 doi:10.1161/STROKEAHA.108.524686; 10.1161/STROKEAHA.108.524686.

19 Fassbender K, Grotta JC, Walter S, et al. Mobile stroke units for prehospital thrombolysis, triage, and beyond: benefits and challenges. *Lancet Neurol* 2017;16:227-37 doi:S1474-4422(17)30008-X [pii].

20 Meretoja A, Strbian D, Mustanoja S, et al. Reducing in-hospital delay to 20 minutes in stroke thrombolysis. *Neurology* 2012;79(4):306-13 doi:10.1212/WNL.0b013e31825d6011.

21 Lahr MM, van der Zee DJ, Vroomen PC, et al. Thrombolysis in acute ischemic stroke: a simulation study to improve pre- and in-hospital delays in community hospitals. *PLoS One* 2013;8:e79049 doi:10.1371/journal.pone.0079049 [doi].

22 Monks T, Pitt M, Stein K, et al. Maximizing the Population Benefit From Thrombolysis in Acute Ischemic Stroke: A Modeling Study of In-Hospital Delays. *Stroke* 2012;43(10):2706-11 doi:10.1161/STROKEAHA.112.663187.

23 Lahr MM, van der Zee DJ, Luijckx GJ, et al. Centralising and optimising decentralised stroke care systems: a simulation study on short-term costs and effects. *BMC Med Res Methodol* 2017;17:5,016-0275-3 doi:10.1186/s12874-016-0275-3 [doi].

24 Lahr M, van der Zee D, Luijckx G, et al.
A simulation based approach for improving utilization of thrombolysis in acute brain infarction. *Medical Care* 2013;51(12):1101-5.

For peer review only

25 Wahlgren N, Ahmed N, Davalos A, et al. Thrombolysis with alteplase for acute ischaemic stroke in the Safe Implementation of Thrombolysis in Stroke-Monitoring Study (SITS-MOST): an observational study. *Lancet* 2007;369:275-82 doi:10.1016/S0140-6736(07)60149-4.

26 Law AM. ExpertFit Version 8 User's Guide. Tuscon, Arizona: Averill M. Law & Associates 2011.

27 Salisbury HR, Banks BJ, Footitt DR, et al. Delay in presentation of patients with acute stroke to hospital in Oxford. *QJM* 1998;91:635-40.

28 Carroll C, Hobart J, Fox C, et al. Stroke in Devon: knowledge was good, but action was poor. *J Neurol Neurosurg Psychiatry* 2004;75:567-71.

29 Barsan WG, Brott TG, Broderick JP, et al. Urgent therapy for acute stroke. Effects of a stroke trial on untreated patients. *Stroke* 1994;25:2132-7.

30 Chen NC, Hsieh MJ, Tang SC, et al. Factors associated with use of emergency medical services in patients with acute stroke. *Am J Emerg Med* 2013;31:788-91 doi:10.1016/j.ajem.2013.01.019 [doi].

31 Powers WJ, Rabinstein AA, Ackerson T, et al. 2018 Guidelines for the Early Management of Patients With Acute Ischemic Stroke: A Guideline for Healthcare Professionals From the American Heart Association/American Stroke Association. *Stroke* 2018;49:e46-e110 doi:10.1161/STR.0000000000000158 [doi].

32 Fonarow GC, Smith EE, Saver JL, et al. Timeliness of tissue-type plasminogen activator therapy in acute ischemic stroke: patient characteristics, hospital factors, and outcomes associated with door-to-needle times within 60 minutes. *Circulation* 2011;123:750-8 doi:10.1161/CIRCULATIONAHA.110.974675.

33 Zinkstok SM, Beenen LF, Luitse JS, et al. Thrombolysis in Stroke within 30 Minutes: Results of the Acute Brain Care Intervention Study. *PLoS One* 2016;11:e0166668 doi:10.1371/journal.pone.0166668 [doi].

34 Meretoja A, Weir L, Ugalde M, et al. Helsinki model cut stroke thrombolysis delays to 25 minutes in Melbourne in only 4 months. *Neurology* 2013;81(12):1071-6 doi:10.1212/WNL.0b013e3182a4a4d2.

35 Lees KR, Bluhmki E, von Kummer R, et al. Time to treatment with intravenous alteplase and outcome in stroke: an updated pooled analysis of ECASS, ATLANTIS, NINDS, and EPITHET trials. *Lancet* 2010;375:1695-703 doi:10.1016/S0140-6736(10)60491-6.

36 Atkins DL, Everson-Stewart S, Sears GK, et al. Epidemiology and outcomes from out-of-hospital cardiac arrest in children: the Resuscitation Outcomes Consortium Epistry-Cardiac Arrest. *Circulation* 2009;119:1484-91 doi:10.1161/CIRCULATIONAHA.108.802678.

37 Jauch EC, Saver JL, Adams HP,Jr, et al. Guidelines for the early management of patients with acute ischemic stroke: a guideline for healthcare professionals from the American Heart

For peer review only

Association/American Stroke Association. *Stroke* 2013;44:870-947 doi:10.1161/STR.0b013e318284056a [doi].

38 Acker JE,3rd, Pancioli AM, Crocco TJ, et al. Implementation strategies for emergency medical services within stroke systems of care: a policy statement from the American Heart Association/American Stroke Association Expert Panel on Emergency Medical Services Systems and the Stroke Council. *Stroke* 2007;38:3097-115 doi:10.1161/STROKEAHA.107.186094.

39 Boode B, Welzen V, Franke C, et al. Estimating the number of stroke patients eligible for thrombolytic treatment if delay could be avoided. *Cerebrovasc Dis* 2007;23:294-8 doi:10.1159/000098330.

40 Lahr MM, van der Zee DJ, Luijckx GJ, et al. Improving acute stroke services in the Netherlands. *BMJ* 2014;348:g3957 doi:10.1136/bmj.g3957 [doi].

41 Monks T, Pitt M, Stein K, et al. Hyperacute stroke care and NHS England's business plan. *BMJ* 2014;348:g3049 doi:10.1136/bmj.g3049 [doi].

42 Chia NH, Leyden JM, Newbury J, et al. Determining the Number of Ischemic Strokes Potentially Eligible for Endovascular Thrombectomy: A Population-Based Study. *Stroke* 2016;47:1377-80 doi:10.1161/STROKEAHA.116.013165 [doi].

43 Caminiti C, Schulz P, Marcomini B, et al. Development of an education campaign to reduce delays in pre-hospital response to stroke. *BMC Emerg Med* 2017;17:20,017-0130-9 doi:10.1186/s12873-017-0130-9 [doi].

44 Table book 2018. Emergency care Netherlands. Zwolle, September 25, 2019. *Accessed October 1, 2019.*

Figure legends:

Figure 1. Acute stroke pathway: description of activities.

For peer review only

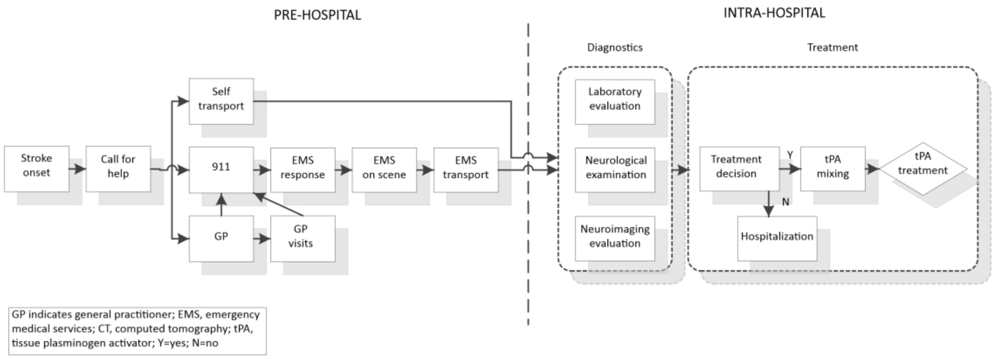
PRE-HOSPITAL
INTRA-HOSPITAL
Diagnostics
Treatment
Stroke onset
Call for help
Self transport
911
EMS response
EMS on scene
EMS transport
GP
GP visits
Laboratory evaluation
Neurological examination
Neuroimaging evaluation
Treatment decision
Y
N
tPA mixing
tPA treatment
Hospitalization
GP indicates general practitioner; EMS, emergency medical services; CT, computed tomography; tPA, tissue plasminogen activator; Y=yes; N=no

Figure 1

Supplementary file

Optimizing acute stroke care organization: a simulation study to assess the potential to increase intravenous thrombolysis rates and patient gains

Maarten M.H. Lahr Ph.D., Durk-Jouke van der Zee Ph.D., Gert-Jan Luijckx M.D., Ph.D.,

Erik Buskens M.D., Ph.D.

Introduction

The main body of text in the manuscript summarizes and discusses the most important results of the study. This supplementary file provides further details on the simulation modeling methodology that was used, the process map underlying the simulation model, model data, and input parameters characterizing scenarios studied.

Simulation modeling methodology

Monte Carlo simulation

The simulation model built conforms to the notion of Monte Carlo simulation.[1, 2] The model represents the acute stroke pathway as it evolves over time by a representation in which state variables change instantaneously at separate, i.e. discrete, points in time.[3] Variety in patient characteristics, activity durations, and medical decision making concerning diagnosis, and intravenous thrombolysis (IVT) treatment are incorporated into the model by probability distributions derived from real system (patient) data. Activity durations include possible patient queueing times, if any, as the stroke patients' urgency usually allows them to queue

For peer review only

jump, effectively rendering waiting times for intra hospital services to remain negligible . Moreover, given their low numbers stroke patients have very limited impact on queueing behavior along the pathway.[4]

Distribution fitting

Probability distributions associated with patient characteristics, and activity durations were determined (fitted) using ExpertFit[5] (Table S1). Main steps concerned:

- Importing real system (patient) data into ExpertFit.
- Fitting theoretical distributions by using the method of maximum likelihood.[3]
- Seeking further evidence in case of a “no fit”, in an attempt to underpin the choice for a specific theoretical distribution. Evidence considered includes conceptual usage of the candidate distribution(s), commonalities between highest ranked distributions, and consultation of domain experts.[6] If such evidence is not found an empirical distribution was chosen.

Set-up of experiments

All experiments concern observations on 10,000 hypothetical patients. The number of patients is chosen such that the 95% confidence interval half width is below 1% of the mean treatment rate.

Software

Plant Simulation was used to model the stroke pathway.[7] Choice of probability distributions and their respective parameters is made using ExpertFit.[5]

For peer review only

Model - process map and data

Model set-up conforms to description of the stroke pathway (Main text, Figure 1). Further details are provided in the process map (Figure S1) and the overview of distributions of time delays and diagnostic characteristics (Table S1). All time delays are expressed in minutes.

Patients are classified according to their route, i.e., mode of transportation towards the hospital:

(1) Emergency Medical Services (EMS): Assumes patients being transported to the hospital by EMS. A patient can be in Route 1 with a probability of 76%. If the patient is in Route 1, then the following quantities need to be simulated for modelling pre-hospital activities: the time from symptom onset to call for help, the choice and time delay at the first responder (i.e. either the general practitioner or 911), the level of urgency set for EMS transport, the time between 911 activation and arrival of the ambulance at the location of the patient, the time spent by ambulance personnel at the location of the patient, and the time required to transport the patient to the hospital. Three levels of urgency for EMS transport are distinguished, i.e., A1, A2, and B. They indicate normative values for ambulance arrival within 15, 30, and > 30 minutes from the 911 call until arrival at the location of the patients. Urgency levels impact EMS response time, time spent on scene and transport time. Intrahospital activities assume the following quantities to be simulated: the time from hospital arrival to neurological examination, the time required for neuroimaging (Computed Tomography, CT scan), the time to laboratory examination of patient blood samples, the time to reach a decision on patient treatment, and the time it takes to mix thrombolytics.

A small group of patients (2% of overall population) in route 1 is initially transported to a community hospital offering no stroke services, thereby facing significant time losses. As

For peer review only

such patients never become eligible for treatment in the real system no quantities were simulated, except for their arrival at the Emergency Department (ED).

(2) Self-transport: Assumes patients not being transported to the hospital by EMS. Instead patients or family/bystanders take care of transportation. A patient can be in Route 2 with a probability of 21%. We simplified the model with respect to the inclusion of patients in Route 2. As none of these patients appeared eligible for treatment in the real system, no quantities were simulated, except for their arrival at the ED. Note how Table S1 clarifies that all patients in this route, except for two, who arrived way beyond the period of 4.5 hours after stroke onset, for whom thrombolysis treatment has been found to be effective.

(3) In-hospital patients: Patients suffering a stroke while being hospitalized. A patient can be in route 3 with a probability of 3%. If the patient is in Route 3 only intra-hospital time delays need to simulated, see route 1.

Next, traversing each route entails sampling from distributions specifying respective activity durations. Note how activity durations may be moderated by diagnostic outcomes. Finally, cumulative delay for a patient is used as an input for the treatment decision. Here a larger delay implies a smaller chance of being treated.

For peer review only

Figure S1. Process map acute stroke pathway.

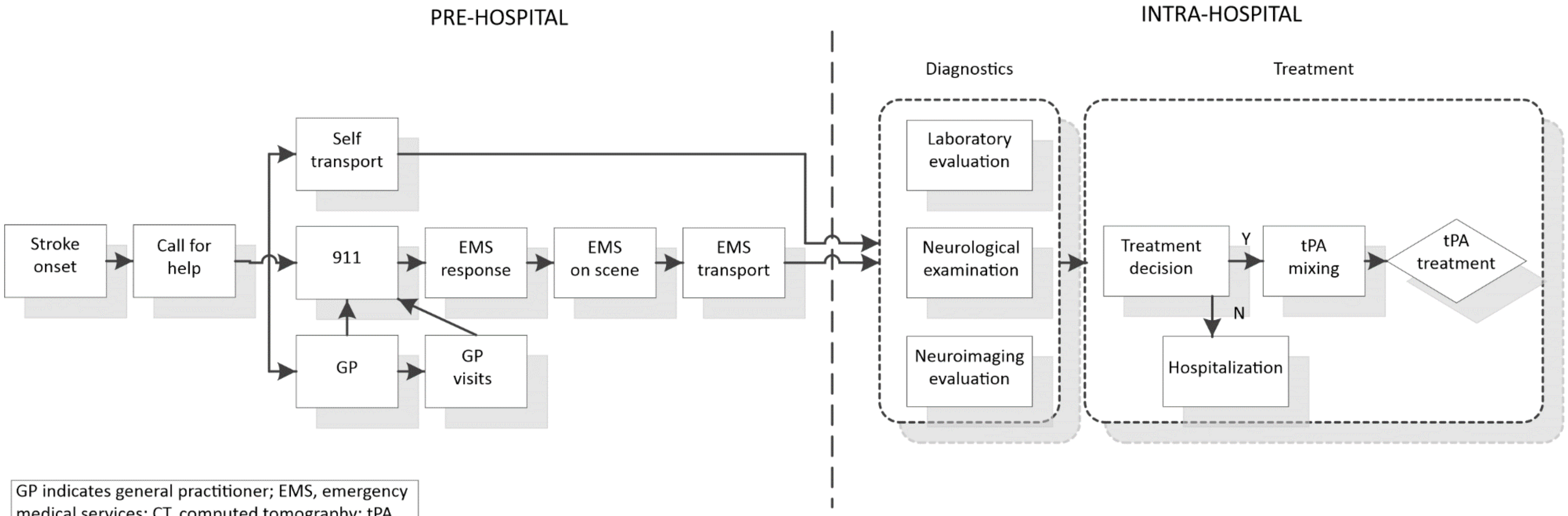

GP indicates general practitioner; EMS, emergency medical services; CT, computed tomography; tPA, tissue plasminogen activator; Y=yes; N=no

Treatment decision

The efficacy of intravenous tissue plasminogen activator in acute brain infarction is greater the earlier it is administered, and the clinical benefit declines progressively over 4.5 hours after stroke onset.[8] For the simulation model the likelihood of treatment is approximated by a linear function, see Figure S2. We used a linear regression model (Y-axis intercept 97.5; slope -0.33) to approximate the chance of IVT treatment set against the overall process time for all patients arriving < 4.5 hours from the onset of stroke symptoms (i.e. eligible for IVT treatment).

Input parameters

Scenarios studied are modeled by changing input parameters, see Table S2 for an overview. Choices of input parameter settings reflecting scenarios are shown in Table S1. Alternative settings of input parameters, in terms of modifications to the distributions underlying the baseline scenario, are shown in the most right column of Table S1. References underlying choice of distributions can be found in the main text (Table 2).

For peer review only

Table S1. Distributions and parameters of time delays and diagnostic characteristics.

Activity duration (minutes)	Base line scenario				Alternative scenarios
	**Distribution**	**Distribution parameters**			**Modifications of baseline distributions**
Time from stroke onset to call for help	Continuous empirical				Scenario 1A,B,C (patient responsiveness): Distribution parameters are adjusted by a factor equal to the quotient of the respective median response times reported for best practices, i.e., 30 (1A), 15 (1B), and 0 minutes (1C), and the baseline scenario (41 minutes).
		Left bound	Right bound	Frequency	
Route 1		0	5	34	
		5	10	4	
		10	15	8	
		15	30	13	
		30	45	15	
		45	60	13	
		60	120	19	
		120	180	13	
		180	240	9	
		240	480	12	
		480	2880	73	

For peer review only

Route 2		120	180	1	
		240	480	1	
		480	2880	58	
Route 3		0	5	6	
Delay first responder					Scenario 5 (expediting response of first responder): Response time is set to zero.
911 call	Uniform	Min (1.00), Max (2.00)			
GP consult by telephone	Uniform	Min (2.00), Max (5.00)			
GP consult by visit	Triangle	Mode (40.00), Min (10.00), Max (30.00)			
Emergency Medical Services					
Response time					Scenario 7A,7B (use of MSU): Response time is set to zero.
A1	Gamma	Alpha (1.36), Beta (6.29)			
A2	Lognormal	Mean (14.21), Standard deviation (6.51)			
B	Beta	Alpha 1 (1.70), Alpha 2 (3.54), a (0.81), b (110.47)			
Time spent on scene					Scenario 6A,B,C (expediting on-scene times): an upper boundary is imposed on distribution outcomes of 15 (6A), 10 (6B) and 0 minutes (6C)
A1	Gamma	Alpha (2.84), Beta (7.42)			
A2	Lognormal	Mean (18.11), Standard deviation (8.39)			Scenario 7B (use of MSU): Transport time is set to zero.
B	Lognormal	Mean (14.25), Standard deviation (8.60)			
Transport time					

For peer review only

A1	Weibull	Alpha (1.93), Beta (19.15)			
A2	Weibull	Alpha (1.43), Beta (16.01)			
B	Beta	Alpha 1 (1.32), Alpha 2 (2.56)			
Time to neurological consultation	Continuous empirical	Left bound	Right bound	Frequency	Scenario 8A,B,C,D (expediting intra hospital processes): an upper boundary is imposed on cumulative distributions reflecting intra hospital processes of 30 (8A), 25 (8B), 20 (8C) and 0 (8D) minutes.
		0	0	93	
		0	1	4	
		1	2	7	
		2	5	6	
		5	24	12	
Time to neuroimaging examination	Continuous empirical	Left bound	Right bound	Frequency	
		2	5	28	
		6	10	54	
		11	15	13	
		16	20	10	
		21	30	8	
		31	56	8	
Time to laboratory examination	Erlang	Mean (32.29), Standard deviation (9.26), Location (2.83)			
Decision making	Triangle	Mode (10), Min (5), Max (20)			

For peer review only

IVT mixing	Constant	5		
**Diagnostics**				
Choice of route<br>1. EMS transport<br>2. Self-transport<br>3. Intra-hospital	Discrete empirical	Value<br>1<br>2<br>3	Frequency<br>213<br>60<br>7	Scenario 2A,B (referral by 911, EMS transport): Distribution parameters, i.e., frequencies have been adjusted such that 60% (2A) and 100% (2B) of patients is referred by 911, and next transported by EMS, implying reduced frequencies for patient self-transport, and GP consults.
Choice of first responder if EMS -transport<br>1. 911 call<br>2. GP consult by phone<br>3. GP consult by visit	Discrete empirical	Value<br>1<br>2<br>3	Frequency<br>30<br>19<br>27	
EMS transport, level of urgency<br>911 call<br>1. A1<br>2. A2<br>3. B<br>GP consult by telephone<br>1. A1	Discrete empirical	Value<br><br>1<br>2<br>3<br><br>1	Frequency<br><br>95<br>3<br>2<br><br>88	

For peer review only

2. A2		2	10	
3. B		3	2	
GP consult by visit				
1. A1		1	60	
2. A2		2	33	
3. B		3	7	

Route 1, 2, and 3 indicate patients transported by ambulance, patients arriving at the hospital by self transport, and those patients suffering a stroke while being hospitalized, respectively; GP, general practitioner; A1, A2, B indicate normative values for ambulance arrival within 15, 30, and > 30 minutes from the 911 call until arrival at the location of the patients, respectively; IVT, intravenous thrombolysis; EMS, emergency medical services.

For peer review only

Table S2. Overview of scenarios and input parameters

Scenario	Input parameters
**Baseline**	
0.Current practice (baseline)	-
**Patient responsiveness**	
1.Patient delay<br>A. Reduced to 30 minutes<br>B. Reduced to 15 minutes<br>C. Reduced to 0 minutes	Time from stroke onset to call for help
2. Mode of referral 911<br>A. Transport by EMS increased to 60%<br>B. Transport by EMS increased to 100%	Choice of route, Choice of first responder
3. Combined best practices patient responsiveness	Combines scenarios 1B and 2A
4. Optimised patient responsiveness	Combines scenarios 1C and 2B
**Pathway set-up**	
5. Response time first responders reduced to 0 minutes	Delay first responder
6. On scene time ambulance personnel<br>A. Reduced to 15 minutes<br>B. Reduced to 10 minutes<br>C. Reduced to 0 minutes	Emergency Medical Services – Time spent on scene
7. Prehospital IVT by MSU<br>A. Transportation time reduced to 0 minutes<br>B. All ambulance delays reduced to 0 minutes	Emergency Medical Services – Response Time (7A,7B), Transport time (7B)
8. Door to IVT times<br>A. Reduced to 30 minutes<br>B. Reduced to 25 minutes<br>C. Reduced to 20 minutes<br>D. Reduced to 0 minutes	Time to neurological consultation, Time to neuroimaging examination, Time to laboratory examination, Decision making, IVT Mixing
9. Combined best practices pathway set-up	Combines scenarios 6B, 7A and 8C
10. Optimised pathway set-up	Combines scenarios 5, 6C, 7B, 8D
**Patient responsiveness and pathway set-up**	
11. Combined best practices patient responsiveness and pathway set-up	Combines scenarios 3 and 9
12. Optimised patient responsiveness and pathway set-up	Combines scenarios 4 and 10

For peer review only

Figure S2. Treatment decision: a patient's chance of being treated given the overall process time.

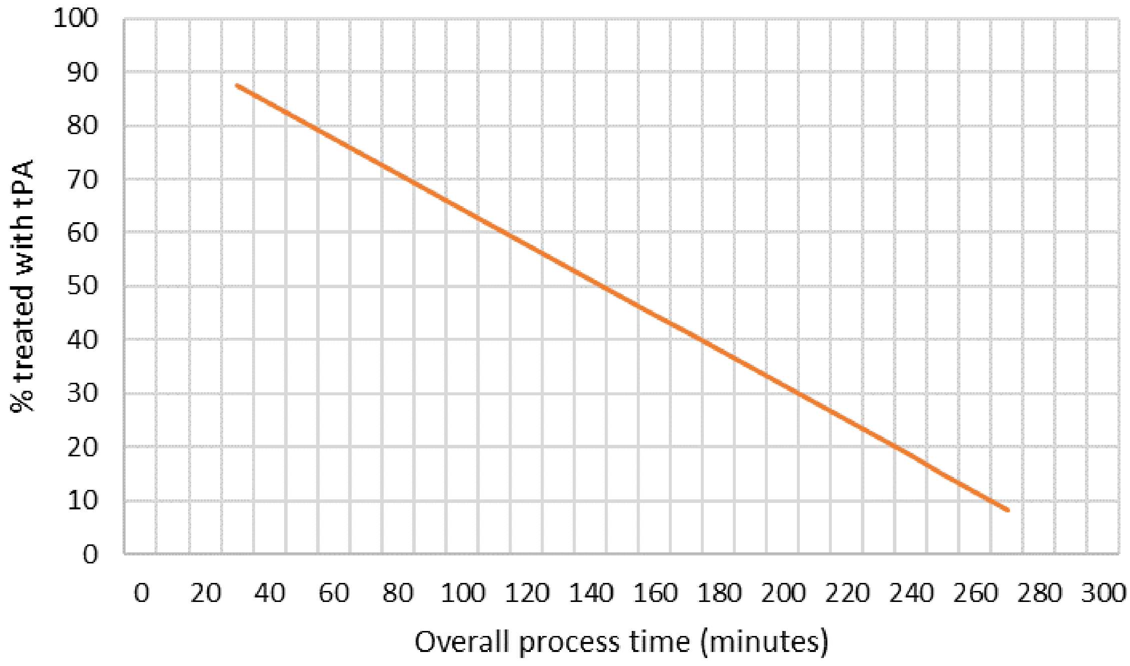

eview only

References

1 Law AM. Simulation Modeling and Analysis: McGrawHill: Boston, 5th edition. 2015.

2 Rubinstein RY. Simulation and the Monte Carlo Method: Wiley: Hoboken, 2nd edition 2008.

3 Law AM, Kelton WD. Simulation modeling and analysis. 4th ed.: McGraw-Hill 2007.

4 Monks, T., van der Zee, D. J., Lahr, M., Allen, M., Pearn, K., James, M. A., Buskens, E., Luijckx, G. J. A framework to accelerate simulation studies of hyperacute stroke systems. *Operations Research for Health Care* 2017;15:57-67 doi:https://doi.org/(...)6/j.orhc.2017.09.002.

5 Law AM. ExpertFit Version 8 User's Guide. Tuscon, Arizona: Averill M. Law & Associates 2011.

6 Stahl JE, Furie KL, Gleason S, et al. Stroke: Effect of implementing an evaluation and treatment protocol compliant with NINDS recommendations. *Radiology* 2003;228:659-68 doi:10.1148/radiol.2283021557.

7 Plant Simulation. Siemens PLM 2012. Available at: http://www.plm.automation.siemens.com/en_us/products/tecnomatix/plant_design/plant_simulation.shtml.

8 Lees KR, Bluhmki E, von Kummer R, et al. Time to treatment with intravenous alteplase and outcome in stroke: an updated pooled analysis of ECASS, ATLANTIS, NINDS, and EPITHET trials. *Lancet* 2010;375:1695-703 doi:10.1016/S0140-6736(10)60491-6.

For peer review only

BMJ Open

Optimizing acute stroke care organization: a simulation study to assess the potential to increase intravenous thrombolysis rates and patient gains

Journal:	*BMJ Open*
Manuscript ID	bmjopen-2019-032780.R2
Article Type:	Original research
Date Submitted by the Author:	04-Dec-2019
Complete List of Authors:	Lahr, Maarten ; University of Groningen, University Medical Center Groningen, Health Technology Assessment, Department of Epidemiology<br>van der Zee, Durk-Jouke; Rijksuniversiteit Groningen Faculteit Economie en Bedrijfskunde, Operations<br>Luijckx, Gert-Jan; University of Groningen, University Medical Center Groningen, Neurology<br>Buskens, Erik; University of Groningen, University Medical Center Groningen, Epidemiology; University of Groningen, Faculty of Economics and Business, Deparment of Operations
**Primary Subject Heading**:	Neurology
Secondary Subject Heading:	Epidemiology, Health services research
Keywords:	Stroke < NEUROLOGY, Organisation of health services < HEALTH SERVICES ADMINISTRATION & MANAGEMENT, EPIDEMIOLOGY

SCHOLARONE™
Manuscripts

Optimizing acute stroke care organization: a simulation study to assess the potential to increase intravenous thrombolysis rates and patient gains

Maarten M.H. Lahr[1] Ph.D., Durk-Jouke van der Zee[2] Ph.D., Gert-Jan Luijckx[3] M.D., Ph.D., Erik Buskens[1,2] M.D., Ph.D.

[1] *Health Technology Assessment, Department of Epidemiology, University of Groningen, University Medical Center Groningen, Groningen, The Netherlands*

[2] *Department of Operations, Faculty of Economics & Business, University of Groningen, Groningen, The Netherlands*

[3] *Department of Neurology, University of Groningen, University Medical Center Groningen, Groningen, The Netherlands*

Corresponding Author:

Maarten M.H. Lahr

University Medical Center Groningen, Department of Epidemiology

P.O. Box 30001. 9700 RB Groningen, The Netherlands

Telephone: + 31 50 361 43 86

E-mail: m.m.h.lahr@umcg.nl

Word count paper: 3490 (max 3500 excl. abstract, references, tables, and figure legends)

Word count abstract: 243 (max 250)

Number of text pages: 13

Number of references: 44 (max 50)

For peer review only

For peer review only - http://bmjopen.bmj.com/site/about/guidelines.xhtml

Number of figures/tables: 4 (max 5 incl. tables)

Brief title: Optimizing acute stroke care systems

Complete author information:

Durk-Jouke van der Zee, PhD. Correspondence address: University of Groningen, Faculty of Economics & Business, Department of Operations. 9747 AE Groningen, The Netherlands. Email: d.j.van.der.zee@rug.nl

Gert-Jan Luijckx, MD, PhD. Correspondence address: University Medical Center Groningen, Department of Neurology. 9713 GZ Groningen, The Netherlands. Email: g.j.luijckx@umcg.nl

Erik Buskens, MP, PhD. Correspondence address: University Medical Center Groningen, Department of Epidemiology. 9713 GZ Groningen, The Netherlands. Email: e.buskens@umcg.nl

Key words: acute stroke, thrombolysis, organizational model, simulation modeling, quality improvement.

Conflict of interest: the authors declare no conflict of interest.

Acknowledgements: None.

For peer review only

Abstract

Objectives To assess potential increases in Intravenous Thrombolysis (IVT) rates given particular interventions in the stroke care pathway.

Design Simulation modeling was used to compare the performance of the current pathway, best practices based on literature review, and an optimized model.

Setting Four hospitals located in the North of the Netherlands, as part of a centralized organizational model.

Participants Ischemic stroke patients prospectively ascertained from February to August 2010.

Intervention The interventions investigated included efforts aimed at patient response and mode of referral, prehospital triage, and intra-hospital delays.

Primary and secondary outcome measures The primary outcome measure was thrombolysis utilization. Secondary measures were onset-treatment time (OTT) and the proportion of patients with excellent functional outcome (mRS 0-1) at 90 days.

Results Of 280 patients with ischemic stroke, 125 (44.6%) arrived at the hospital within 4.5 hours, and 61 (21.8%) received IVT. The largest improvements in IVT treatment rates, OTT, and the proportion of patients with mRS scores of 0-1 can be expected when patient response is limited to 15 minutes (IVT rate +5.8%; OTT -6 minutes; excellent mRS scores +0.2%), door-to-needle time to 20 minutes (IVT rate +4.8%; OTT -28 minutes; excellent mRS scores +3.2%), and 911 calls are increased to 60% (IVT rate +2.9%; OTT -2 minutes; excellent mRS scores +0.2%). The combined implementation of all potential best practices could increase IVT rates by 19.7% and reduce OTT by 56 minutes.

Conclusions Improving IVT rates to well above 30% appears possible if all known best practices are implemented.

Strengths and limitations of the study

For peer review only

- The simulation modelling study included a comprehensive collection of patient level data from both the pre- and intrahospital acute stroke pathway.
- A simulation-based approach, as presented in this paper, can be instrumental in facilitating a broad overview of the set-up and performance of stroke pathways.
- Effects of capacity constraints on patients' waiting for care services are not explicitly modelled given their high priorities, allowing them to queue jump.
- Costs items associated with the proposed interventions could not be collected and controlled for.
- Estimations of time intervals used for model building might have changed over time.

Introduction

For peer review only

Intravenous thrombolysis (IVT) is an effective therapy for acute ischemic stroke up to 4.5 hours after the onset of symptoms.[1, 2] This therapy is substantially underused, however, with 8-10% of all stroke patients worldwide currently receiving IVT.[3, 4] In contrast, treatment rates of up to 35% have been achieved in optimized settings.[5, 6] The organization of stroke care is an important factor in realizing timely hospital arrival and treatment.[7, 8] The centralization of care at designated stroke centers has been demonstrated to increase the proportion of patients arriving at the hospital in time for acute treatment.[9-11] Given the substantial decrease in the benefit of treatment with increasing time delays, further efforts to expedite hospital arrival and subsequent treatment remain of crucial importance.[12, 13]

Various studies have investigated factors associated with efficiency in each part of the acute stroke pathway. Although it has been generally established that delay on the part of patients and/or bystanders is a primary factor in delaying hospital arrival,[14] interventions aimed at improving optimal response by calling 911 immediately have exhibited varying success, and many lack sustained implementation[15] (as is the case in other domains of medicine as well).[16] Ambulance transportation to hospitals that offer acute treatment is associated with shorter onset-to-door times, reductions in intra-hospital delays, and increases in treatment rates.[15, 17, 18] The provision of acute treatment by a Mobile Stroke Unit (MSU) before hospital arrival has been identified as a promising method for reducing time to IVT. [19]Another widely studied topic concerns reducing the time between hospital arrival and treatment (door-to-needle time, or DTN), with reported DTN times as low as 20 minutes.[20]

The aforementioned studies have demonstrated clear benefits in terms of time saved. They nevertheless lack a broader overview of pathway set-up and its performance. Instead of addressing the solution space as a whole, they target isolated elements of the stroke pathway. = The lack of a broader overview is due in part to the predominant use of randomized controlled trials (RCT) as the main research vehicle. Given the effort involved in their set-up, RCT studies

For peer review only

focus predominantly on separate and singular elements of pathway performance. They may therefore be less suitable for investigating complex care systems (e.g., acute stroke treatment). In particular, timely hospital arrival and the treatment of acute stroke patients relies on a series of intertwined activities concerning patient diagnostics and transportation.

One potential alternative methodology is simulation modeling. Proceeding from a detailed description of both pre-hospital and intra-hospital time delays and diagnostics, an accurate representation of pathway performance can be developed *in silico*, including the validation of IVT rates, time to treatment, patient outcomes (as measured by the modified Rankin Scale or mRS), and other clinically relevant outcome measures.[21, 22] This approach would allow the examination of all time delays and diagnostic steps, thereby providing clinicians and policymakers with an accurate overview of obstacles currently existing within care pathways. In addition, scenarios for hypothetical approaches to improvement based on clinical guidelines, literature observations, and/or expert opinion can be modeled and studied for their cumulative effects on relevant clinical outcome measures.[23, 24]

The aims of this simulation-modeling study were (1) to estimate the cumulative potential for improving IVT utilization by implementing best practices on the organization of the stroke pathway and, subsequently, (2) to explore areas in which further improvement is needed in order to achieve a fully optimized setting, in addition to identifying obstacles to such optimization.

Methods

This article is based on a six-month, prospective, multi-center study performed in a centralized organizational stroke-care setting in the north of the Netherlands from February through July 2010.[9] Patient-level data were collected on time delays and diagnostics, thereby providing detailed insight into patient flow and potential obstacles in both the pre-hospital and intra-

For peer review only

hospital pathways (Table 1). A schematic overview of patient flow and the steps included in the analyses is presented in Figure 1.

Table 1. Descriptive statistics of activity durations and diagnostics

Number of patients	280
Age in years (SD)	70 (14)
Male (%)	156 (56)
**Patient responsiveness**	
Time from symptom onset to call for help, valid cases (%)	152 (54)
Median, minutes (IQR)	41 (5-130)
Mode of referral (%)	
General practitioner	129 (46)
911	84 (30)
Self-referral	60 (21)
In-hospital patients	7 (3)
**Pathway set-up**	
Transported by EMS (%)	213 (76)
Median response time, min (IQR)	9 (7-12)
Median on scene time, min (IQR)	20 (15-25)
Median transportation time, min (IQR)	17 (9-22)
Median time from hospital arrival to neurological examination, min (range)	2 (0-15)
Median time from hospital arrival to CT examination min (IQR)	12 (6-15)
Median time from hospital arrival to laboratory examination, min (IQR)	32 (27-37)
Median door to IVT time, min (IQR)	35 (25-45)

SD indicates standard deviation; IQR, Interquartile Range; EMS, Emergency Medical Services; CT, Computed Tomography; IVT, Intravenous Thrombolysis.

Setting and participants

The centralized organizational setting consisted of four hospitals in the northern region of the Netherlands, with IVT being administered only in the University Medical Center Groningen (UMCG). Together with the other three community hospitals, general practitioner (GP) offices, and emergency medical services (EMS), arrangements were made to transport presumed stroke

For peer review only

victims immediately to the UMCG, thus bypassing community hospitals that might have been located closer to the patient's location. The international protocol for IVT (adjusted ECASS III[25]) and the regional protocol for pre-hospital management were followed. The region addressed in this study comprises approximately 580,000 inhabitants, with a population density of 250 inhabitants per square kilometer. The study population consisted of ischemic stroke patients admitted to all four hospitals between February and August 2010. Case ascertainment was confirmed by the final hospital discharge diagnosis of ischemic stroke, thereby excluding stroke mimics.

The data collected included information on time delays and diagnostics along the entire stroke chain. Stroke care pathways can be described in several distinct phases: hyperacute (emergency), acute and rehabilitation. Within this study we focused on the hyperacute phase, ranging from symptom onset until acute treatment with IVT. Time delays included the time from symptom onset to call for help, delay at first response (GP or EMS services), EMS transport times, and intra-hospital diagnostics, which included time from hospital arrival to neurological examination, Computed Tomography (CT) scan, laboratory testing, and IVT.

Simulation model

The current study was performed using a previously validated simulation model.[24] More detailed information on simulation modeling methodology, input parameters, model and model data used may be found in a supplementary file. This model was populated with patient-level data from a previous observational study.[9] The model was validated by comparing IVT treatment percentages and OTT to those reported in the observational study. The next step consisted of developing scenarios in which alternative pathway set-ups, associated time delays, and diagnostics were imputed based on literature observations, clinical guidelines, and expert opinion. Using the simulation model, hypothetical patients were passed through the system,

For peer review only

estimating the impact of intervening at various points in the acute stroke pathway. Interventions were modeled by changing the underlying statistical distributions to redistribute patients across time delays and diagnostics.[26]

Scenarios

We used simulation modeling to investigate the effects of changing pathway set-ups, based on three models: (1) a baseline model of acute stroke care; (2) a model reflecting best practices, based on a review of the current literature, clinical guidelines, and expert opinion; and (3) an optimized model. Interventions were selected according to the obstacles identified in the current centralized organizational model. Obstacles within the study setting included delayed emergency response by patients following symptom onset, mode of referral (GP or 911), time spent on the scene by ambulance personnel, and intra-hospital delays. The simulation model was used to perform hypothetical interventions in the pathway to calculate clinically relevant outcomes.[24] The outcomes were compared to the baseline performance of the current system to estimate the potential for improvement, to optimize system performance, and to identify obstacles that have yet to be overcome.

<u>Baseline</u>

The baseline model describes the performance of the centralized organizational model for acute stroke care, as described in the previous observational study.[9] A description of time delays and diagnostics along each step of the pathway is provided in Table 1.

<u>Best practice</u>

The scenarios and input parameters that were investigated are described in Table 2.

For peer review only - http://bmjopen.bmj.com/site/about/guidelines.xhtml

Patient responsiveness: We estimated the relative impact of reducing the time between stroke onset and call for help by patients, their families, and/or bystanders by adjusting the distribution of their response times by a factor equal to the quotient of the respective median response times reported for best practices (15-30 minutes)[27, 28] and the baseline scenario (41 minutes).

Mode of referral: We modeled a scenario in which patients, their families, and/or bystanders predominantly (60%) chose 911 as the mode of entry.[29, 30]

Time spent on the scene by ambulance personnel: In this scenario, we modeled the time spent on the scene by ambulance personnel following a 911 call by imposing an upper boundary on on-scene delay. Based on the guidelines of the American Stroke Association, the time spent on the scene should be no more than 15 minutes.[31] We also modeled the implementation of a "scoop and run protocol," which includes prompt transport (≤ 10 minutes) with initial management efforts, while postponing elaborate triage.

Pre-hospital treatment by MSU: In this scenario, we modeled pre-hospital IVT provided by a MSU by adjusting only the transport time of the ambulance. Assuming that the MSU would be stationed at the hospital, we considered only the transportation time from the hospital to the patient scene.

For peer review only

Table 2. Overview of scenarios and input parameters.

Factor	Baseline	Scenarios	Study	Input parameters
**Patient responsiveness**				
1. Patient delay	41 minutes*	A. Reduced to 30 minutes (*best practice*)<br>B. Reduced to 15 minutes (*best practice*)<br>C. Reduced to 0 minutes (*optimised*)	Salisbury et al. (1998)<br>Carrol et al. (2004)	Time from stroke onset to call for help
2. Mode of referral 911	30%	A. Transport by EMS increased to 60% (*best practice*)<br>B. Transport by EMS increased to 100% (*optimised*)	Barsan et al. (1994),Chen et al. (2013)	Choice of route, choice of first responder
3. Combined best practices patient responsiveness		Combines scenarios 1B and 2A		See factors 1 and 2
4. Optimised patient responsiveness		Combines scenarios 1C and 2B		See factors 1 and 2
**Pathway set-up**				
5. Response time first responders	9 minutes*	Response time first responders reduced to 0 minutes (*optimised*)		Delay first responder
6. On scene time ambulance personnel	20 minutes*	A. Reduced to 15 minutes (*best practice*)<br>B. Reduced to 10 minutes (*best practice*)<br>C. Reduced to 0 minutes (*optimised*)	Jauch et al. (2013), Acker et al. (2007)<br>Atkins et al. (2009)	Emergency Medical Services – Time spent on scene
7. Prehospital IVT by MSU Transportation time	17 minutes*	A. Transportation time reduced to 0 minutes (*best practice*)<br>B. All ambulance delays reduced to 0 minutes (*optimised*)	Fassbender et al. (2017)	Emergency Medical Services – Response time (7A,7B), Emergency Medical Services – Transport Time (7B)
8. Door to IVT times	35 minutes*	A. Reduced to 30 minutes (*best practice*)<br>B. Reduced to 25 minutes (*best practice*)<br>C. Reduced to 20 minutes (*best practice*)<br>D. Reduced to 0 minutes (*optimised*)	Zinkstok et al. (2016)<br>Meretoja et al. (2013)<br>Meretoja et al. (2012)	Time to neurological consultation, Time to neuroimaging examination, Time to laboratory examination, Decision making, IVT Mixing
9. Combined best practices pathway set-up		Combines scenarios 6B, 7A and 8C		See factors 6, 7 and 8
10. Optimised pathway set-up		Combines scenarios 5, 6C, 7B and 8D		See factors 5, 6, 7 and 8
**Patient responsiveness and pathway set-up**				
11. Combined best practices patient responsiveness and pathway set-up		Combines scenarios 3 and 9		See factors 3 and 9
12. Optimised patient responsiveness and pathway set-up		Combines scenarios 4 and 10		See factors 4 and 10

EMS indicates emergency medical services; IVT, intravenous thrombolysis; MSU, Mobile Stroke Unit.
* Median

For peer review only

Intra-hospital processes: Intra-hospital delays comprise all activities performed between arrival at the hospital until the start of IVT (door-to-needle time; see Figure 1). Based on the guidelines of the American Stroke Association, door-to-needle time should be no more than 60 minutes.[32] Evidence from clinical practice suggests that DTN times within a range of 20 to 30 minutes are attainable.[20, 33, 34]

Combined best-practice scenarios

Three scenarios were performed in which best practices were combined for patient responsiveness and pathway set-up. Patient responsiveness was modeled by combining patient response with the mode of referral following stroke onset. Pathway set-up was modeled by combining pre-hospital and intra-hospital best practices. A third scenario combines best practices for patient responsiveness and pathway set-up.

Optimized scenarios

Additional scenarios were defined to interpret findings on the effects of the implementation of best practices (or combinations thereof) by generating upper boundaries to pathway performance, thereby building on unrealistically optimistic assumptions. For the optimized scenarios, we extrapolated best practices by setting the parameter for associated time delays to 0 or by setting the diagnostic quality parameter to 100%.

Outcome measures

The primary outcome measure was the proportion of patients treated with IVT. Secondary outcome measures were the total process time (onset-to-treatment time), IVT within various time intervals (0-90, 91-180, and 181-270 minutes), the proportion of patients with favorable

For peer review only

outcomes at 90 days (modified Rankin Scale 0-1), and additional healthy life days (calculated using OTT estimates).[12, 35]

Analysis

For each of the scenarios described above, we calculated new hypothetical IVT treatment rates and secondary outcome measures, based on the number of patients arriving in time for acute treatment at the hospital (i.e., within 4 hours after symptom onset), the number treated with IVT, and the time to treatment. Chi-square tests were used to compare categorical variables.

Ethics approval and patient consent

Informed consent was obtained from all subjects participating in the prospective study[9] and extended for the current simulation study. No additional approval was necessary because for this simulation study an anonymized dataset was used.

Data availability statement

The authors state that anonymized data will be shared by request from any qualified investigator.

Patient and public involvement

Patients and public were not involved in the design of this study. For this modelling study non-identifiable patient data was used. Study results will be disseminated through poster presentations and publication in peer-reviewed journals.

Results

For peer review only

This study reflects the experiences of 280 ischemic stroke patients referred to the UMCG and three community hospitals, as part of a centralized organizational model. Baseline and demographic characteristics are described in Table 1. In all, 125 (44.6%) patients arrived at the hospital within 4.5 hours, 61 (21.8%) received IVT, and the median OTT was 127 minutes. Of the patients receiving IVT, 17.0% were treated within 90 minutes of symptom onset. Patient delay, intra-hospital delays, and mode of referral (GP or 911) were identified as the greatest obstacles to receiving IVT (Table 1).

Simulation experiments

The results of the simulation experiments are presented in Table 3.

Patient responsiveness: If patients had contacted emergency services sooner (i.e., within 30 and 15 minutes), up to 27.6% (CI 26.7% – 28.4%) of the total population would have been treated with IVT (Table 3, Scenarios 1A and 1B), and the OTT would have been reduced to 122 minutes (CI 121 – 124).[27, 28]

Assuming a patient delay of 0 minutes (Table 3, Scenario 1C), 64.0% of the total population would have been treated with IVT, and the OTT would have been reduced to 92 minutes (CI 91 – 92).

Mode of referral: Assuming 60% of all patients contacting 911 immediately following stroke onset (Table 3, Scenario 2A) increased the IVT rate to 24.6% (CI 23.8% – 25.5%) and reduced the OTT to 127 minutes (CI 125 – 128).[29]

If all patients (100%) had called 911 (Table 3, Scenario 2B), the IVT rates would have increased further to 28.4% (CI 27.5% – 29.3%), and the OTT would have been reduced to 124 minutes (CI 123 – 126).

For peer review only

Time spent on the scene by ambulance personnel: Shortening the time spent on the scene to 15 and 10 minutes (Table 3, Scenarios 6A and 6B),[36] increased the IVT treatment rate up to 23.3% (CI 22.4% – 24.1%) and reduced the OTT to 121 minutes (CI 120 – 123).[37, 38]

For peer review only

Table 3. Re-configuration centralised model: results simulation experiments.

	IVT rate (95% CI)	OTT minutes (95% CI)	IVT 0-1.5 hours	IVT 1.5-3.0 hours	IVT 3.0-4.5 hours	mRS 0-1†	Extra healthy days‡
**Scenario**							
0.Current practice	21.8% (20.9% - 22.6%)	129 (127 – 130)	17.0%	70.9%	12.1%	12.5%	
**Patient responsiveness**							
1.Patient delay							
A. Reduced to 30 minutes	23.7% (22.9% - 24.6%)	127 (125 – 129)	16.9%	72.2%	10.9%	12.6%	2.9
B. Reduced to 15 minutes	27.6% (26.7% - 28.4%)*	122 (121 – 124)	16.5%	76.7%	6.8%	12.7%	11.3
C. Reduced to 0 minutes	64.0% (63.0% - 64.9%)*	92 (91 – 92)	45.7%	54.1%	0.2%	16.2%	66.9
2. Mode of referral 911							
A. Increased to 60%	24.6% (23.8% - 25.5%)	127 (125 – 128)	18.8%	70.0%	11.2%	12.8%	3.9
B. Increased to 100%	28.4% (27.5% - 29.3%)*	124 (123 – 126)	18.3%	71.4%	10.3%	12.7%	7.8
3. Combined best practices patient responsiveness (1B+2A)	31.0% (30.1% - 31.9%)*	120 (118 – 121)	19.2%	74.4%	6.4%	13.0%	16.3
4. Optimised patient responsiveness (1C + 2B)	64.3% (63.3% - 65.2%)*	98 (97 – 98)	36.2%	63.7%	0.1%	15.1%	55.7
**Pathway set-up**							
5. Response time first responders reduced to 0 minutes	23.3% (22.4% - 24.1%)	121 (119 - 122)	21.8%	68.9%	9.3%	13.2%	14.4
6. On scene time ambulance personnel							
A. Reduced to 15 minutes	22.8% (21.9% - 23.6%)	124 (122 – 126)	19.4%	69.9%	10.7%	12.8%	8.6
B. Reduced to 10 minutes	23.3% (22.4% - 24.1%)	121 (120 – 123)	21.7%	68.5%	9.8%	13.1%	13.4
C. Reduced to 0 minutes	24.7% (23.8% - 25.5%)	114 (112 – 116)	31.4%	60.4%	8.2%	14.3%	26.3
7. Prehospital IVT by MSU							
A. Transportation time reduced to 0 minutes	23.2% (22.4% - 24.0%)	121 (120 – 123)	22.8%	67.4%	9.8%	13.2%	13.1
B. All ambulance delays reduced to 0 minutes	25.6% (24.7% – 26.4%)	109 (107 – 110)	38.6%	54.3%	7.0%	15.1%	35.8
8. Door to IVT times							
A. Reduced to 30 minutes	25.2% (24.3% - 26.0%)	110 (108 – 111)	34.3%	57.7%	8.0%	14.6%	34.4
B. Reduced to 25 minutes	25.9% (25.0% - 26.8%)	106 (104 – 107)	39.3%	53.2%	7.5%	15.2%	41.5
C. Reduced to 20 minutes	26.6% (25.7% - 27.4%)	101 (99 – 103)	44.2%	48.8%	7.0%	15.7%	49.8
D. Reduced to 0 minutes	29.8% (28.9% - 30.7%)*	83 (81 – 85)	62.9%	32.1%	5.0%	17.9%	81.9
9. Combined best practices pathway set-up (6B+7A+8C)	30.8% (29.9% - 31.7%)*	80 (79 – 82)	68.9%	26.3%	4.8%	18.6%	87.3
10. Optimised pathway set-up (5 + 6C + 7B + 8D)	38.5% (37.6% - 39.5%)*	39 (37 – 40)	86.0%	11.3%	2.7%	20.6%	161.7
**Patient responsiveness and pathway set-up**							

11. Combined best practices patient responsiveness and pathway set-up (3+9)	41.5% (40.5% - 42.4%)*	73 (72 – 74)	77.4%	20.5%	2.2%	19.6%	99.9
12. Optimised patient responsiveness and pathway set-up (4+10)	97.7% (97.4% – 98.0%)*	0 (0 – 0)	100.0%	0.0%	0.0%	22.2%	231.6

IVT indicates intravenous thrombolysis; CI, confidence interval; OTT, onset-treatment-time; tPA indicates tissue plasminogen activator; mRS, modified rankin scale; MSU, mobile stroke unit.

* Sig. <0.05

† Indicates the proportion of patients with good outcome (mRS 0-1) ascribed to treatment with thrombolysis.[33]

‡ Indicated the number of additional days in healthy life by minute reduction in OTT.[1]

For peer review only

Reducing time on the scene to 0 minutes resulted in a projected IVT rate of 24.7% (CI 23.8% – 25.5%) (Table 3, Scenario 6C) and a decrease in the OTT to 114 minutes (CI 112 – 116).

Pre-hospital treatment by Mobile Stroke Unit (MSU): In this set-up, 23.2% (CI 22.4% – 24.0%) of patients would have been treated with IVT (Table 3, Scenario 7A) and OTT would have been reduced to 121 minutes (CI 120 – 123).

The elimination of both the response time and transportation time of the MSU (Table 3, Scenario 7B) resulted in a projected 25.6% (CI 24.7% - 26.4%) of patients that could have received IVT and a reduction of the OTT to 109 minutes (CI 107 – 110).

Intra-hospital processes: If the DTN time had been shortened to a maximum of 30 and 20 minutes (Table 3, Scenarios 8A-C), up to 26.6% (CI 25.7% - 27.4%) of the total population would have been treated with IVT, and the OTT would have been reduced to 101 minutes (CI 99 – 103).

If the DTN had been reduced to 0 minutes (Table 3, Scenario 8D), 29.8% (CI 28.9 – 30.7%) of all patients would have been treated with IVT, and the OTT would have been reduced to 83 minutes (CI 81 – 85).

Combined best practice scenarios

Combining best practices for patient responsiveness and pathway set-up (Table 3, scenario's 3,9,11) resulted in up to 41.5% (CI 40.5% - 42.4%) of all patients being treated with IVT and reduced the OTT to 73 minutes (CI 72 – 74).

Optimized scenarios

Assuming optimized patient responsiveness (i.e. all patients calling 911 immediately following stroke onset; Table 3, Scenario 4) resulted in 64.3% of the total population (CI 63.3% – 65.2%)

For peer review only

being treated with IVT and reduced the OTT to 98 minutes (CI 97 – 98). The optimization of pathway set-up (Table 3, Scenario 10) resulted in 38.5% (CI 37.6% – 39.5%) of all patients receiving IVT and reduced the OTT to 39 minutes (CI 37 – 40). The combination of all optimized scenarios (Table 3, Scenario 12) resulted in a cumulative total of 97.7% (CI 97.4% - 98.0%) of all patients being treated with IVT and reduced the OTT to 0 minutes.

Discussion

This study demonstrates that IVT treatment rates above 30% would be possible if best practices were to be implemented within our setting. We modeled several scenarios to generate insight into the potential for quality improvements in our acute stroke chain of care. Although improvements in patient responsiveness would yield the largest potential gains within our pathway, even modest changes in this regard are likely to be challenging and costly to achieve.[16] In contrast, improvements in other areas (e.g., intra-hospital delays and time spent by ambulance personnel at the patient's location) might be easier to achieve and would still lead to clinically relevant increases in IVT rates. As indicated in previous studies, even small reductions in time to treatment with IVT are associated with considerable increases in the length of healthy life, and they may require only relatively simple organizational changes involving minimal effort at little or no cost.[12]

The results of our study may be useful as a guide for prioritizing interventions along the acute stroke pathway and for estimating their potential impact on the effectiveness of the pathway. A simulation-based approach, as presented in this paper, can be instrumental in facilitating a broad overview of the set-up and performance of stroke pathways. This could provide clinicians and policymakers with speedy answers – at little effort or cost – concerning how new or widely advocated practices could be used to improve their pathways, thus allowing them to direct investments to the interventions that matter most. It thus has the potential to

For peer review only

replace RCT studies or serve as a precursor to a focused RCT, which could be scoped as the net result of a simulation approach.

In our study, we observed the greatest effects on IVT rates, time to treatment, and patient outcomes after improving the responsiveness of patients and/or bystanders by reducing the time from symptom onset to the call for help, thereby expediting intra-hospital care services and by increasing the number of 911 calls made by patients or bystanders. In contrast, a scenario in which pre-hospital transportation delays were reduced by the implementation of a MSU resulted in only moderate effects. Combining all of the best-practice scenarios resulted in a maximum of 41.5% of patients being treated with IVT. This is substantially higher than current benchmark figures on clinical practice, which suggest a maximum IVT rate of around 35%.[6, 39]

Proceeding from a scenario in which best practices have been implemented, remaining challenges include realizing further decreases in time delays in both the pre-hospital and intra-hospital phases. The feasibility of such initiatives in clinical practice might be limited in the short term, however, given the current lack of evidence concerning solutions for further expediting care and logistics services at reasonable costs. For example, the goal of reducing time spent on the scene by ambulance personnel to less than 10 minutes or reducing door-to-needle time to less than 20 minutes would probably be unrealistic, given the need to handle and observe the patient, to complete diagnostic tests, and to interpret findings. Although further improvements in the proportion of patients calling 911 directly following symptom onset could potentially result in further increases in IVT rates, they would also necessitate large-scale and repetitive publicity campaigns comparable to those launched to raise public awareness on stroke symptoms and how to act.

The organizational model for acute stroke care delivery is currently receiving a great deal of attention in the Netherlands, as well as beyond.[40, 41] The emergence of endovascular treatment (EVT) for patients facing large-vessel occlusions has opened up a whole new

For peer review only

dimension in terms of acute stroke pathway set-up and patient logistics. Following IVT, eligible patients must now undergo additional diagnostic evaluation (e.g., CT angiography and perfusion CT), followed by such EVT treatment modalities as groin punctures and initial attempt at clot retrieval with the device up to the angioseal following successful recanalization. In addition, within the current "drip-and-ship" treatment paradigm, eligible patients may initially be admitted to community hospitals before being transferred to comprehensive stroke centers with EVT capacity, thereby further increasing the number of logistical steps. Given the time-sensitive nature of acute stroke interventions, this extension of the pathway necessitates the re-organization of acute stroke care within regions and settings. In this respect, simulation modeling could facilitate insight into the complex interplay of separate elements of the pathway. Currently positioned as a follow-up treatment by current guidelines, availability of EVT does not change the need for optimizing utilization of IVT, nor does it impact the acute stroke pathway set-up for IVT. Moreover, the subgroup of patients eligible for EVT is relatively small, around 7% of all stroke patients.[42]

Our study is subject to several limitations. First, our simulation models did not consider the response of GPs when contacted as first responders. Although this has been signaled as an issue for delays in hospital arrival for patients,[43] no studies on best practices were identified in the literature. Second, because the costs and cost-effectiveness associated with pathway improvements in our setting were not estimated in our model, it was not possible to control for them. Third, the results of our findings might not be generalizable to other settings, due to the unique position of the UMCG, which serves as a stroke center in a centralized organizational model deployed within its region. As noted in a previous publication, however, the generic modeling approach adopted in this study could be extended to include a description of a decentralized organizational model, which receives IVT candidates within its own catchment areas.[23] Also, because patients were enrolled in the observational study back in 2010 these

For peer review only

results will not fully reflect current practice. However, review of internal databases show that IVT treatment percentages have remained largely stable over the last years, fluctuating around 25% with a DTN time of around 35 minutes. EMS response times have remained constant over the years.[44] In addition, pathway set-up of acute stroke patients receiving IVT remained similar over the years. Finally, model assumptions excluded the possibility for capacity constraints influencing patient waiting times, as patients and/or transport queuing seldom occurs due to the high prioritization that potential acute stroke patients receive throughout the pathway. However, we acknowledge that such constraints might occur in other stroke care systems. Future activities should be aimed at extending the simulation-based approach to include the drip-and-ship model currently employed in acute stroke treatment (e.g., EVT).

Conclusions

The results of this study indicate that the cumulative effects of implementing best practices on the organization of stroke care would clearly exceed current benchmarks for treatment rates. Remaining obstacles might be difficult to overcome given the limited availability of solution to further expedite care and logistical services at tolerable costs. A broader overview facilitated by simulation is suggested as instrumental in supporting decision-makers and clinicians in their efforts to evaluate the set-up and performance of acute stroke pathways.

Contributor statement ML and DJZ designed the study, performed the experiments and analyzed the data. ML drafted the manuscript, DJZ, GJL and EB critically revised the manuscript for intellectual content and approved the final version of the manuscript for publication.

Competing interests None declared

For peer review only

Funding None

Data sharing statement

The data used and analysed in the current study is available from the corresponding author upon request.

References

1 . Tissue plasminogen activator for acute ischemic stroke. The National Institute of Neurological Disorders and Stroke rt-PA Stroke Study Group. *N Engl J Med* 1995;333:1581-7 doi:10.1056/NEJM199512143332401.

2 Hacke W, Kaste M, Bluhmki E, et al. Thrombolysis with alteplase 3 to 4.5 hours after acute ischemic stroke. *N Engl J Med* 2008;359:1317-29 doi:10.1056/NEJMoa0804656.

3 Bauer A, Limburg M, Visser MC. Variation in clinical practice of intravenous thrombolysis in stroke in the Netherlands. *Cerebrovasc Dis Extra* 2013;3:74-7 doi:10.1159/000350707; 10.1159/000350707.

4 Adeoye O, Hornung R, Khatri P, et al. Recombinant tissue-type plasminogen activator use for ischemic stroke in the United States: a doubling of treatment rates over the course of 5 years. *Stroke* 2011;42:1952-5 doi:10.1161/STROKEAHA.110.612358.

5 Waite K, Silver F, Jaigobin C, et al. Telestroke: a multi-site, emergency-based telemedicine service in Ontario. *J Telemed Telecare* 2006;12:141-5 doi:10.1258/135763306776738611.

6 Stolz E, Hamann GF, Kaps M, et al. Regional differences in acute stroke admission and thrombolysis rates in the German federal state of Hesse. *Dtsch Arztebl Int* 2011;108:607-11 doi:10.3238/arztebl.2011.0607 [doi].

7 Bekelis K, Marth NJ, Wong K, et al. Primary Stroke Center Hospitalization for Elderly Patients With Stroke: Implications for Case Fatality and Travel Times. *JAMA Intern Med* 2016;176:1361-8 doi:10.1001/jamainternmed.2016.3919 [doi].

8 Willeit J, Geley T, Schoch J, et al. Thrombolysis and clinical outcome in patients with stroke after implementation of the Tyrol Stroke Pathway: a retrospective observational study. *Lancet Neurol* 2015;14:48-56 doi:10.1016/S1474-4422(14)70286-8 [doi].

9 Lahr MM, Luijckx GJ, Vroomen PC, et al. Proportion of patients treated with thrombolysis in a centralized versus a decentralized acute stroke care setting. *Stroke* 2012;43:1336-40 doi:10.1161/STROKEAHA.111.641795.

10 Ramsay AI, Morris S, Hoffman A, et al. Effects of Centralizing Acute Stroke Services on Stroke Care Provision in Two Large Metropolitan Areas in England. *Stroke* 2015;46:2244-51 doi:10.1161/STROKEAHA.115.009723 [doi].

For peer review only

11 Morris S, Hunter RM, Ramsay AI, et al. Impact of centralising acute stroke services in English metropolitan areas on mortality and length of hospital stay: difference-in-differences analysis. *BMJ* 2014;349:g4757 doi:10.1136/bmj.g4757 [doi].

12 Meretoja A, Keshtkaran M, Saver JL, et al. Stroke thrombolysis: save a minute, save a day. *Stroke* 2014;45:1053-8 doi:10.1161/STROKEAHA.113.002910 [doi].

13 Tsivgoulis G, Katsanos AH, Kadlecova P, et al. Intravenous thrombolysis for ischemic stroke in the golden hour: propensity-matched analysis from the SITS-EAST registry. *J Neurol* 2017;264:912-20 doi:10.1007/s00415-017-8461-8 [doi].

14 Kleindorfer D, Khoury J, Broderick JP, et al. Temporal trends in public awareness of stroke: warning signs, risk factors, and treatment. *Stroke* 2009;40:2502-6 doi:10.1161/STROKEAHA.109.551861 [doi].

15 Fassbender K, Balucani C, Walter S, et al. Streamlining of prehospital stroke management: the golden hour. *Lancet Neurol* 2013;12:585-96 doi:10.1016/S1474-4422(13)70100-5; 10.1016/S1474-4422(13)70100-5.

16 Wakefield MA, Loken B, Hornik RC. Use of mass media campaigns to change health behaviour. *Lancet* 2010;376:1261-71 doi:10.1016/S0140-6736(10)60809-4.

17 Evenson KR, Foraker RE, Morris DL, et al. A comprehensive review of prehospital and in-hospital delay times in acute stroke care. *Int J Stroke* 2009;4:187-99 doi:10.1111/j.1747-4949.2009.00276.x.

18 Rose KM, Rosamond WD, Huston SL, et al. Predictors of time from hospital arrival to initial brain-imaging among suspected stroke patients: the North Carolina Collaborative Stroke Registry. *Stroke* 2008;39:3262-7 doi:10.1161/STROKEAHA.108.524686; 10.1161/STROKEAHA.108.524686.

19 Fassbender K, Grotta JC, Walter S, et al. Mobile stroke units for prehospital thrombolysis, triage, and beyond: benefits and challenges. *Lancet Neurol* 2017;16:227-37 doi:S1474-4422(17)30008-X [pii].

20 Meretoja A, Strbian D, Mustanoja S, et al. Reducing in-hospital delay to 20 minutes in stroke thrombolysis. *Neurology* 2012;79(4):306-13 doi:10.1212/WNL.0b013e31825d6011.

21 Lahr MM, van der Zee DJ, Vroomen PC, et al. Thrombolysis in acute ischemic stroke: a simulation study to improve pre- and in-hospital delays in community hospitals. *PLoS One* 2013;8:e79049 doi:10.1371/journal.pone.0079049 [doi].

22 Monks T, Pitt M, Stein K, et al. Maximizing the Population Benefit From Thrombolysis in Acute Ischemic Stroke: A Modeling Study of In-Hospital Delays. *Stroke* 2012;43(10):2706-11 doi:10.1161/STROKEAHA.112.663187.

23 Lahr MM, van der Zee DJ, Luijckx GJ, et al. Centralising and optimising decentralised stroke care systems: a simulation study on short-term costs and effects. *BMC Med Res Methodol* 2017;17:5,016-0275-3 doi:10.1186/s12874-016-0275-3 [doi].

For peer review only

24 Lahr M, van der Zee D, Luijckx G, et al. A simulation based approach for improving utilization of thrombolysis in acute brain infarction. *Medical Care* 2013;51(12):1101-5.

25 Wahlgren N, Ahmed N, Davalos A, et al. Thrombolysis with alteplase for acute ischaemic stroke in the Safe Implementation of Thrombolysis in Stroke-Monitoring Study (SITS-MOST): an observational study. *Lancet* 2007;369:275-82 doi:10.1016/S0140-6736(07)60149-4.

26 Law AM. ExpertFit Version 8 User's Guide. Tuscon, Arizona: Averill M. Law & Associates 2011.

27 Salisbury HR, Banks BJ, Footitt DR, et al. Delay in presentation of patients with acute stroke to hospital in Oxford. *QJM* 1998;91:635-40.

28 Carroll C, Hobart J, Fox C, et al. Stroke in Devon: knowledge was good, but action was poor. *J Neurol Neurosurg Psychiatry* 2004;75:567-71.

29 Barsan WG, Brott TG, Broderick JP, et al. Urgent therapy for acute stroke. Effects of a stroke trial on untreated patients. *Stroke* 1994;25:2132-7.

30 Chen NC, Hsieh MJ, Tang SC, et al. Factors associated with use of emergency medical services in patients with acute stroke. *Am J Emerg Med* 2013;31:788-91 doi:10.1016/j.ajem.2013.01.019 [doi].

31 Powers WJ, Rabinstein AA, Ackerson T, et al. 2018 Guidelines for the Early Management of Patients With Acute Ischemic Stroke: A Guideline for Healthcare Professionals From the American Heart Association/American Stroke Association. *Stroke* 2018;49:e46-e110 doi:10.1161/STR.0000000000000158 [doi].

32 Fonarow GC, Smith EE, Saver JL, et al. Timeliness of tissue-type plasminogen activator therapy in acute ischemic stroke: patient characteristics, hospital factors, and outcomes associated with door-to-needle times within 60 minutes. *Circulation* 2011;123:750-8 doi:10.1161/CIRCULATIONAHA.110.974675.

33 Zinkstok SM, Beenen LF, Luitse JS, et al. Thrombolysis in Stroke within 30 Minutes: Results of the Acute Brain Care Intervention Study. *PLoS One* 2016;11:e0166668 doi:10.1371/journal.pone.0166668 [doi].

34 Meretoja A, Weir L, Ugalde M, et al. Helsinki model cut stroke thrombolysis delays to 25 minutes in Melbourne in only 4 months. *Neurology* 2013;81(12):1071-6 doi:10.1212/WNL.0b013e3182a4a4d2.

35 Lees KR, Bluhmki E, von Kummer R, et al. Time to treatment with intravenous alteplase and outcome in stroke: an updated pooled analysis of ECASS, ATLANTIS, NINDS, and EPITHET trials. *Lancet* 2010;375:1695-703 doi:10.1016/S0140-6736(10)60491-6.

36 Atkins DL, Everson-Stewart S, Sears GK, et al. Epidemiology and outcomes from out-of-hospital cardiac arrest in children: the Resuscitation Outcomes Consortium Epistry-Cardiac Arrest. *Circulation* 2009;119:1484-91 doi:10.1161/CIRCULATIONAHA.108.802678.

For peer review only

37 Jauch EC, Saver JL, Adams HP,Jr, et al. Guidelines for the early management of patients with acute ischemic stroke: a guideline for healthcare professionals from the American Heart Association/American Stroke Association. *Stroke* 2013;44:870-947 doi:10.1161/STR.0b013e318284056a [doi].

38 Acker JE,3rd, Pancioli AM, Crocco TJ, et al. Implementation strategies for emergency medical services within stroke systems of care: a policy statement from the American Heart Association/American Stroke Association Expert Panel on Emergency Medical Services Systems and the Stroke Council. *Stroke* 2007;38:3097-115 doi:10.1161/STROKEAHA.107.186094.

39 Boode B, Welzen V, Franke C, et al. Estimating the number of stroke patients eligible for thrombolytic treatment if delay could be avoided. *Cerebrovasc Dis* 2007;23:294-8 doi:10.1159/000098330.

40 Lahr MM, van der Zee DJ, Luijckx GJ, et al. Improving acute stroke services in the Netherlands. *BMJ* 2014;348:g3957 doi:10.1136/bmj.g3957 [doi].

41 Monks T, Pitt M, Stein K, et al. Hyperacute stroke care and NHS England's business plan. *BMJ* 2014;348:g3049 doi:10.1136/bmj.g3049 [doi].

42 Chia NH, Leyden JM, Newbury J, et al. Determining the Number of Ischemic Strokes Potentially Eligible for Endovascular Thrombectomy: A Population-Based Study. *Stroke* 2016;47:1377-80 doi:10.1161/STROKEAHA.116.013165 [doi].

43 Caminiti C, Schulz P, Marcomini B, et al. Development of an education campaign to reduce delays in pre-hospital response to stroke. *BMC Emerg Med* 2017;17:20,017-0130-9 doi:10.1186/s12873-017-0130-9 [doi].

44 Table book 2018. Emergency care Netherlands. Zwolle, September 25, 2019. *Accessed October 1, 2019.*

Figure legends:

Figure 1. Acute stroke pathway: description of activities.

For peer review only

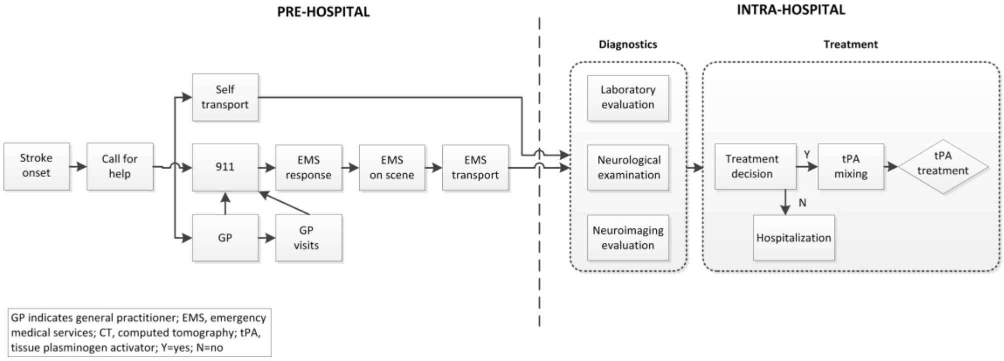

Figure 1

227x81mm (300 x 300 DPI)

Supplementary file

Optimizing acute stroke care organization: a simulation study to assess the potential to increase intravenous thrombolysis rates and patient gains

Maarten M.H. Lahr Ph.D., Durk-Jouke van der Zee Ph.D., Gert-Jan Luijckx M.D., Ph.D., Erik Buskens M.D., Ph.D.

Introduction

The main body of text in the manuscript summarizes and discusses the most important results of the study. This supplementary file provides further details on the simulation modeling methodology that was used, the process map underlying the simulation model, model data, and input parameters characterizing scenarios studied.

Simulation modeling methodology

<u>Monte Carlo simulation</u>

The simulation model built conforms to the notion of Monte Carlo simulation.[1, 2] The model represents the acute stroke pathway as it evolves over time by a representation in which state variables change instantaneously at separate, i.e. discrete, points in time.[3] Variety in patient characteristics, activity durations, and medical decision making concerning diagnosis, and intravenous thrombolysis (IVT) treatment are incorporated into the model by probability distributions derived from real system (patient) data. Activity durations include possible patient queueing times, if any, as the stroke patients' urgency usually allows them to queue

For peer review only

jump, effectively rendering waiting times for intra hospital services to remain negligible . Moreover, given their low numbers stroke patients have very limited impact on queueing behavior along the pathway.[4]

Distribution fitting

Probability distributions associated with patient characteristics, and activity durations were determined (fitted) using ExpertFit[5] (Table S1). Main steps concerned:

- Importing real system (patient) data into ExpertFit.
- Fitting theoretical distributions by using the method of maximum likelihood.[3]
- Seeking further evidence in case of a “no fit”, in an attempt to underpin the choice for a specific theoretical distribution. Evidence considered includes conceptual usage of the candidate distribution(s), commonalities between highest ranked distributions, and consultation of domain experts.[6] If such evidence is not found an empirical distribution was chosen.

Set-up of experiments

All experiments concern observations on 10,000 hypothetical patients. The number of patients is chosen such that the 95% confidence interval half width is below 1% of the mean treatment rate.

Software

Plant Simulation was used to model the stroke pathway.[7] Choice of probability distributions and their respective parameters is made using ExpertFit.[5]

For peer review only

Model - process map and data

Model set-up conforms to description of the stroke pathway (Main text, Figure 1). Further details are provided in the process map (Figure S1) and the overview of distributions of time delays and diagnostic characteristics (Table S1). All time delays are expressed in minutes.

Patients are classified according to their route, i.e., mode of transportation towards the hospital:

(1) Emergency Medical Services (EMS): Assumes patients being transported to the hospital by EMS. A patient can be in Route 1 with a probability of 76%. If the patient is in Route 1, then the following quantities need to be simulated for modelling pre-hospital activities: the time from symptom onset to call for help, the choice and time delay at the first responder (i.e. either the general practitioner or 911), the level of urgency set for EMS transport, the time between 911 activation and arrival of the ambulance at the location of the patient, the time spent by ambulance personnel at the location of the patient, and the time required to transport the patient to the hospital. Three levels of urgency for EMS transport are distinguished, i.e., A1, A2, and B. They indicate normative values for ambulance arrival within 15, 30, and > 30 minutes from the 911 call until arrival at the location of the patients. Urgency levels impact EMS response time, time spent on scene and transport time. Intrahospital activities assume the following quantities to be simulated: the time from hospital arrival to neurological examination, the time required for neuroimaging (Computed Tomography, CT scan), the time to laboratory examination of patient blood samples, the time to reach a decision on patient treatment, and the time it takes to mix thrombolytics.

A small group of patients (2% of overall population) in route 1 is initially transported to a community hospital offering no stroke services, thereby facing significant time losses. As

For peer review only

such patients never become eligible for treatment in the real system no quantities were simulated, except for their arrival at the Emergency Department (ED).

(2) Self-transport: Assumes patients not being transported to the hospital by EMS. Instead patients or family/bystanders take care of transportation. A patient can be in Route 2 with a probability of 21%. We simplified the model with respect to the inclusion of patients in Route 2. As none of these patients appeared eligible for treatment in the real system, no quantities were simulated, except for their arrival at the ED. Note how Table S1 clarifies that all patients in this route, except for two, who arrived way beyond the period of 4.5 hours after stroke onset, for whom thrombolysis treatment has been found to be effective.

(3) In-hospital patients: Patients suffering a stroke while being hospitalized. A patient can be in route 3 with a probability of 3%. If the patient is in Route 3 only intra-hospital time delays need to simulated, see route 1.

Next, traversing each route entails sampling from distributions specifying respective activity durations. Note how activity durations may be moderated by diagnostic outcomes. Finally, cumulative delay for a patient is used as an input for the treatment decision. Here a larger delay implies a smaller chance of being treated.

Timing of stroke onset on the day did not affect our model assumptions, because we did not expect any impact on capacity constraints. Out of office hours were not included because the hospital under study (University Medical Center Groningen) has 24/7/365 occupation of personnel and facilities for acute stroke treatment.

For peer review only

Figure S1. Process map acute stroke pathway.

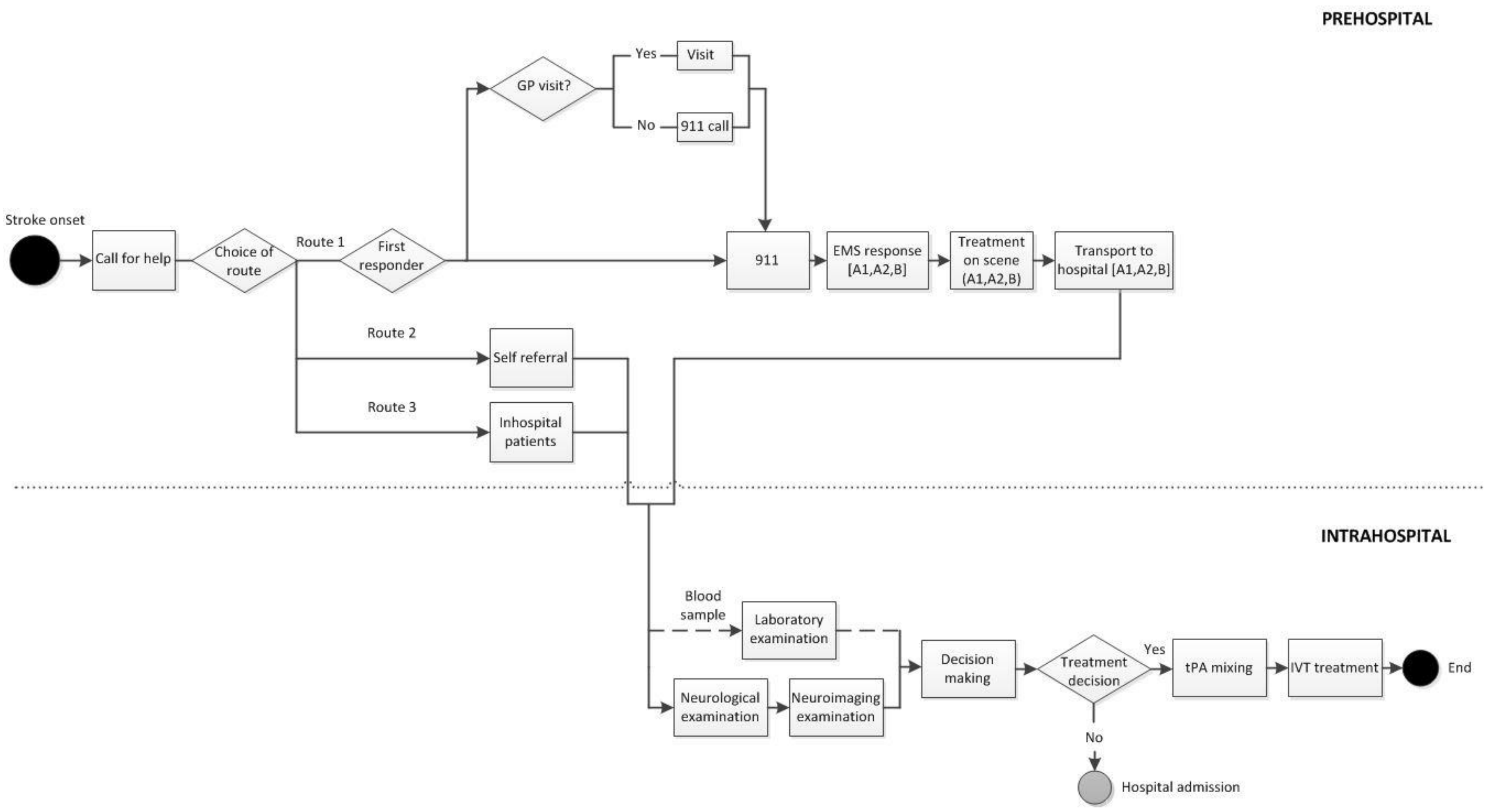

Treatment decision

The efficacy of intravenous tissue plasminogen activator in acute brain infarction is greater the earlier it is administered, and the clinical benefit declines progressively over 4.5 hours after stroke onset.[8] For the simulation model the likelihood of treatment is approximated by a linear function, see Figure S2. We used a linear regression model (Y-axis intercept 97.5; slope -0.33) to approximate the chance of IVT treatment set against the overall process time for all patients arriving < 4.5 hours from the onset of stroke symptoms (i.e. eligible for IVT treatment).

Input parameters

Scenarios studied are modeled by changing input parameters, see Table 2 (main text) for an overview. Choices of input parameter settings reflecting scenarios are shown in Table S1. Alternative settings of input parameters, in terms of modifications to the distributions underlying the baseline scenario, are shown in the most right column of Table S1. References underlying choice of distributions can be found in the main text (Table 2).

For peer review only

Table S1. Distributions and parameters of time delays and diagnostic characteristics.

**Activity duration (minutes)**	**Base line scenario**				**Alternative scenarios**
	**Distribution**	**Distribution parameters**			**Modifications of baseline distributions**
Time from stroke onset to call for help	Continuous empirical	Left bound	Right bound	Frequency (%)	Scenario 1A,B,C (patient responsiveness): Distribution parameters are adjusted by a factor equal to the quotient of the respective median response times reported for best practices, i.e., 30 (1A), 15 (1B), and 0 minutes (1C), and the baseline scenario (41 minutes).
Route 1		0	5	34 (16)	
		5	10	4 (2)	
		10	15	8 (4)	
		15	30	13 (6)	
		30	45	15 (7)	
		45	60	13 (6)	
		60	120	19 (9)	
		120	180	13 (6)	
		180	240	9 (4)	
		240	480	12 (6)	
		480	2880	73 (34)	

For peer review only

Route 2		120	180	1 (2)	
		240	480	1 (2)	
		480	2880	58 (96)	
Route 3		0	5	6 (100)	
Delay first responder					Scenario 5 (expediting response of first responder): Response time is set to zero.
911 call	Uniform	Min (1.00), Max (2.00)			
GP consult by telephone	Uniform	Min (2.00), Max (5.00)			
GP consult by visit	Triangular	Mode (40.00), Min (10.00), Max (30.00)			
Emergency Medical Services					
Response time					Scenario 7A,7B (use of MSU): Response time is set to zero.
A1	Gamma	Alpha (1.36), Beta (6.29)			
A2	Lognormal	Mean (14.21), Standard deviation (6.51)			
B	Beta	Alpha 1 (1.70), Alpha 2 (3.54), a (0.81), b (110.47)			
Time spent on scene					Scenario 6A,B,C (expediting on-scene times): an upper boundary is imposed on distribution outcomes of 15 (6A), 10 (6B) and 0 minutes (6C)
A1	Gamma	Alpha (2.84), Beta (7.42)			
A2	Lognormal	Mean (18.11), Standard deviation (8.39)			Scenario 7B (use of MSU): Transport time is set to zero.
B	Lognormal	Mean (14.25), Standard deviation (8.60)			
Transport time					

For peer review only

A1	Weibull	Alpha (1.93), Beta (19.15)			
A2	Weibull	Alpha (1.43), Beta (16.01)			
B	Beta	Alpha 1 (1.32), Alpha 2 (2.56)			
Time to neurological consultation	Continuous empirical	Left bound	Right bound	Frequency (%)	Scenario 8A,B,C,D (expediting intra hospital processes): an upper boundary is imposed on cumulative distributions reflecting intra hospital processes of 30 (8A), 25 (8B), 20 (8C) and 0 (8D) minutes.
		0	0	93 (76)	
		0	1	4 (3)	
		1	2	7 (6)	
		2	5	6 (5)	
		5	24	12 (10)	
Time to neuroimaging examination	Continuous empirical	Left bound	Right bound	Frequency (%)	
		2	5	28 (23)	
		6	10	54 (44)	
		11	15	13 (11)	
		16	20	10 (8)	
		21	30	8 (7)	
		31	56	8 (7)	
Time to laboratory examination	Erlang	Mean (32.29), Standard deviation (9.26), Location (2.83)			
Decision making	Triangular	Mode (10), Min (5), Max (20)			

For peer review only

IVT mixing	Constant	5		
**Diagnostics**				
Choice of route<br>1. EMS transport<br>2. Self-transport<br>3. Intra-hospital	Discrete empirical	Value<br>1<br>2<br>3	Frequency (%)<br>213 (76)<br>60 (21)<br>7 (3)	Scenario 2A,B (referral by 911, EMS transport): Distribution parameters, i.e., frequencies have been adjusted such that 60% (2A) and 100% (2B) of patients is referred by 911, and next transported by EMS, implying reduced frequencies for patient self-transport, and GP consults.
Choice of first responder if EMS -transport<br>1. 911 call<br>2. GP consult by phone<br>3. GP consult by visit	Discrete empirical	Value<br>1<br>2<br>3	Frequency (%)<br>30 (39)<br>19 (25)<br>27 (36)	
EMS transport, level of urgency<br>911 call<br>1. A1<br>2. A2<br>3. B<br>GP consult by telephone<br>1. A1	Discrete empirical	Value<br><br>1<br>2<br>3<br><br>1	%<br><br>95<br>3<br>2<br><br>88	

For peer review only

2. A2		2	10	
3. B		3	2	
GP consult by visit				
1. A1		1	60	
2. A2		2	33	
3. B		3	7	

Route 1, 2, and 3 indicate patients transported by ambulance, patients arriving at the hospital by self transport, and those patients suffering a stroke while being hospitalized, respectively; GP, general practitioner; A1, A2, B indicate normative values for ambulance arrival within 15, 30, and > 30 minutes from the 911 call until arrival at the location of the patients, respectively; IVT, intravenous thrombolysis; EMS, emergency medical services.

For peer review only

Figure S2. Treatment decision: a patient's chance of being treated given the overall process time.

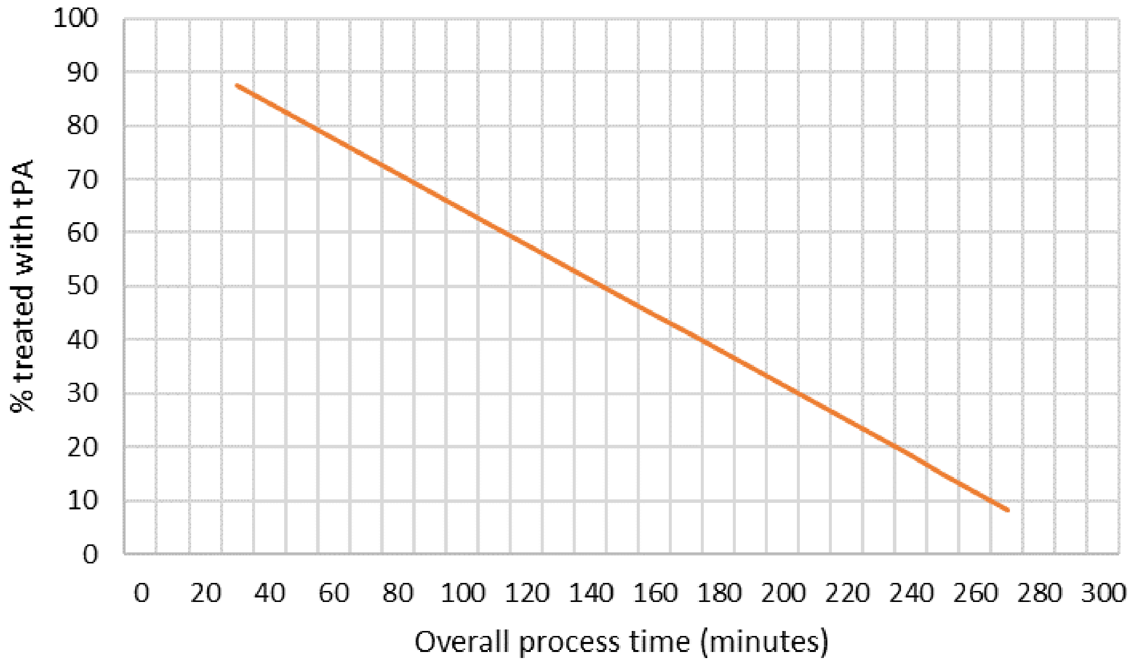

review only

References

1 Law AM. Simulation Modeling and Analysis: McGrawHill: Boston, 5th edition. 2015.

2 Rubinstein RY. Simulation and the Monte Carlo Method: Wiley: Hoboken, 2nd edition 2008.

3 Law AM, Kelton WD. Simulation modeling and analysis. 4th ed.: McGraw-Hill 2007.

4 Monks, T., van der Zee, D. J., Lahr, M., Allen, M., Pearn, K., James, M. A., Buskens, E., Luijckx, G. J. A framework to accelerate simulation studies of hyperacute stroke systems. *Operations Research for Health Care* 2017;15:57-67 doi:https://doi.org/(...)6/j.orhc.2017.09.002.

5 Law AM. ExpertFit Version 8 User's Guide. Tuscon, Arizona: Averill M. Law & Associates 2011.

6 Stahl JE, Furie KL, Gleason S, et al. Stroke: Effect of implementing an evaluation and treatment protocol compliant with NINDS recommendations. *Radiology* 2003;228:659-68 doi:10.1148/radiol.2283021557.

7 Plant Simulation. Siemens PLM 2012. Available at: http://www.plm.automation.siemens.com/en_us/products/tecnomatix/plant_design/plant_simulation.shtml.

8 Lees KR, Bluhmki E, von Kummer R, et al. Time to treatment with intravenous alteplase and outcome in stroke: an updated pooled analysis of ECASS, ATLANTIS, NINDS, and EPITHET trials. *Lancet* 2010;375:1695-703 doi:10.1016/S0140-6736(10)60491-6.

For peer review only
